# A DNA Damage Response System Associated with the phosphoCTD of Elongating RNA Polymerase II

**DOI:** 10.1371/journal.pone.0060909

**Published:** 2013-04-16

**Authors:** Tiffany Sabin Winsor, Bartlomiej Bartkowiak, Craig B. Bennett, Arno L. Greenleaf

**Affiliations:** Department of Biochemistry, Duke Center for RNA Biology, Duke University Medical Center, Durham, North Carolina, United States of America; Tulane University Health Sciences Center, United States of America

## Abstract

RNA polymerase II translocates across much of the genome and since it can be blocked by many kinds of DNA lesions, detects DNA damage proficiently; it thereby contributes to DNA repair and to normal levels of DNA damage resistance. However, the components and mechanisms that respond to polymerase blockage are largely unknown, except in the case of UV-induced damage that is corrected by nucleotide excision repair. Because elongating RNAPII carries with it numerous proteins that bind to its hyperphosphorylated CTD, we tested for effects of interfering with this binding. We find that expressing a decoy CTD-carrying protein in the nucleus, but not in the cytoplasm, leads to reduced DNA damage resistance. Likewise, inducing aberrant phosphorylation of the CTD, by deleting *CTK1*, reduces damage resistance and also alters rates of homologous recombination-mediated repair. In line with these results, extant data sets reveal a remarkable, highly significant overlap between phosphoCTD-associating protein genes and DNA damage-resistance genes. For one well-known phosphoCTD-associating protein, the histone methyltransferase Set2, we demonstrate a role in DNA damage resistance, and we show that this role requires the phosphoCTD binding ability of Set2; surprisingly, Set2’s role in damage resistance does not depend on its catalytic activity. To explain all of these observations, we posit the existence of a CTD-Associated DNA damage Response (CAR) system, organized around the phosphoCTD of elongating RNAPII and comprising a subset of phosphoCTD-associating proteins.

## Introduction

Damage in DNA can block the progression of elongating RNAPII [1]. With the recent understanding that RNAPII transcribes much of the human genome at some level [2], it is clear that RNAPII could play a major role in detecting and signaling the presence of many types of DNA damage in a large fraction of the total DNA of a cell. Because RNAPII elongation complexes stopped at DNA damage are quite stable [1], the polymerase does not have to specifically recognize a DNA lesion in order to detect it efficiently; the RNAPII merely needs to be blocked by it. This mode of finding DNA damage has been referred to as “recognition by proxy” [1].

The best understood pathway of RNA polymerase II-mediated DNA damage recognition and repair is transcription-coupled nucleotide excision repair of UV-induced pyrimidine dimers [3]. In contrast, recognition and repair of other lesions are poorly understood, although accumulating evidence suggests a broad role for the RNAPII transcription elongation complex in responses to multiple kinds of DNA damage (e.g. [4]–[9]). One presumably general feature of recognition by proxy is that the blocked elongation complex itself serves to trigger downstream events [10], [11]. These events can include ubiquitinylation and degradation of RPB1 [12] and, in mammals, activation of the p53-dependent cell cycle checkpoint [11] and even homologous chromosome pairing [7].

The C-terminal repeat domain (CTD) of polymerase subunit RPB1 coordinates many RNAPII-related processes [13]–[16], and it is sensible to predict that it may also coordinate transcription-linked DNA damage responses; indeed, normal damage responses are dependent on RNAPII’s having an intact CTD [17]. In addition, proper phosphorylation of the CTD by CTDK-I (CTD kinase I) is required for normal levels of resistance to several chemical and physical damaging agents in yeast (see Table S1). Moreover, a number of phosphoCTD-associating proteins (PCAPs) are already known to be required for normal resistance to DNA damaging agents or are otherwise involved in DNA repair/genome stability; these include yeast PCAPs Ess1, Hrr25, Chl1, Pms1, Rtt103, Sen1 and TopoI [8], [9], [18]–[21], and metazoan PCAPs PARP1, TopoI, RecQ5 and ASF/SF2 [22]–[27]. Finally, deletions of genes for any one of the three CTDK-I subunits (Ctk1, Ctk2 or Ctk3) are synthetically lethal with individual deletions of a large number of “DNA integrity” genes (see Table S2). These interactions imply functional relationships between CTDK-I and numerous repair proteins, including those involved in homologous recombination (HR)-mediated repair.

Beyond studies of individual proteins, genome-wide screens have expanded the panorama of components affecting levels of resistance to DNA damaging agents [28]. While most screens examined haploid yeast to find DNA damage “resistance” genes (deletions thereof cause sensitivity to the damage), a few screens have exploited the now-available diploid deletion strain collection, and these screens have identified many genes not previously known to be involved in DNA damage resistance [29]–[32]. A number of the genes identified in these studies encode proteins now known to associate with the phosphoCTD of elongating RNAPII, hinting at undescribed links between responses to DNA damage and PCTD-associating components of the RNAPII transcription elongation complex.

In this report we describe approaches aimed at elucidating connections between the PCTD of elongating RNA polymerase II and response to DNA damage. Our results indicate that resistance to several DNA damaging agents and repair of certain DNA lesions require a normally phosphorylated CTD and its proper associations with PCTD-associating proteins. To explain these results and other extant observations we proffer the CTD-Associated DNA damage Response (CAR) system, organized around the phosphoCTD of elongating RNAPII and incorporating a significant subset of phosphoCTD-associating proteins.

## Results

### Disrupting Deployment of PCAPs to the PCTD Leads to Damage Sensitivity

**Figure 1:**
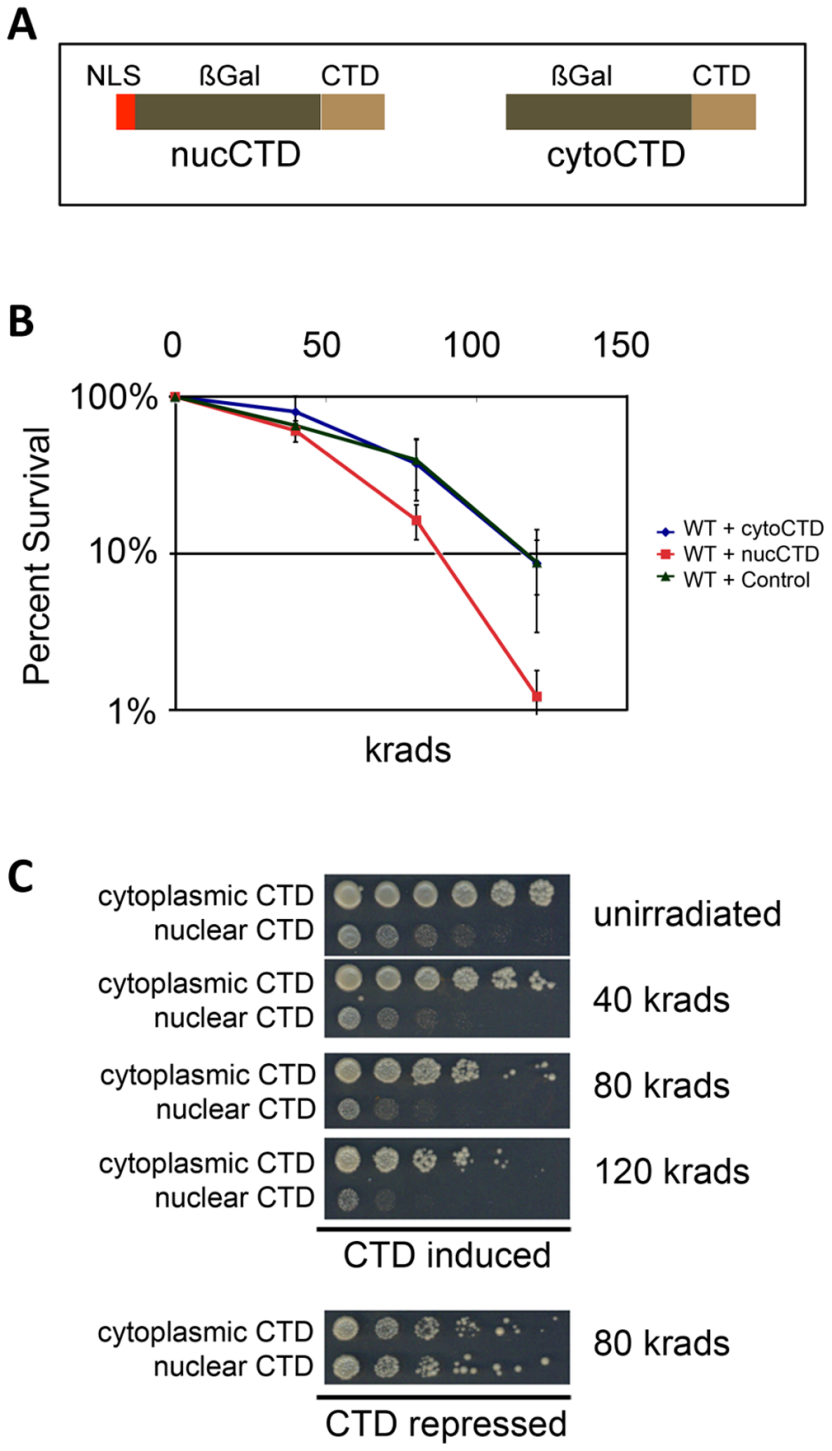
Interfering with binding of PCAPs to the CTD of elongating RNAPII leads to ionizing radiation sensitivity. *(A)* Diagrams of CTD-carrying fusion proteins expressed under inducing conditions (in galactose medium) either in the nucleus (nucCTD) or in the cytoplasm (cytoCTD). *(B)* Percent survival of yeast cells as a function of radiation dose. Liquid cultures of the three strains (three isolates of each in galactose medium) were grown well into stationary phase (G0) and exposed to a single dose of gamma-rays of the indicated intensities. Aliquots were then plated to galactose containing medium and survival was determined by counting colonies after several days at 30°C. Bars indicate standard deviations. *(C)* Growth of 5-fold serial dilutions expressing nucCTD or cytoCTD after exposure to ionizing radiation.

We sought to test the importance of PCAP associations with the PCTD of elongating RNAPII for their role in DNA damage resistance. As a first test, we disrupted the normal deployment of PCAPs to the PCTDs of transcribing polymerases and checked whether this resulted in increased sensitivity to DNA damaging agents. To disrupt normal PCAP deployment, we made use of two expression constructs in which a full-length yeast CTD is fused C-terminally to a ⅔-length *E. coli* ß-galactosidase either containing or not containing a nuclear localization signal (“nucCTD” and “cytoCTD,” respectively) (Fig. 1A and Fig. S1); expression of the fusion proteins is regulated by a *GAL* promoter and thus is repressed by glucose and induced by galactose. We predicted that overexpressed nucCTD protein would enter the nucleus, become hyperphosphorylated, compete for PCAP/CAR protein binding with elongating RNAPII, and render the cells sensitive to DNA damage. In contrast, the cytoCTD protein would remain in the cytoplasm, presumably not interfering with PCAP-RNAPII interactions in the nucleus, and damage sensitivity would not be affected.

After determining that the two Gal-induced fusion proteins were expressed at similar levels, in the correct cellular compartment and phosphorylated (Fig. S1), we analyzed their effects on sensitivity to ionizing radiation (IR)-induced DNA damage. Galactose induction led to a 10-fold decrease in survival rate for the strain expressing the nucCTD fusion protein, relative to the strains expressing either the cytoCTD fusion protein or no fusion protein (Fig. 1B & C). In contrast, all three glucose-grown (repressed) strains were indistinguishable in survival rates (e.g. Fig. 1C). We also found that nucCTD-expressing cells were more sensitive to the chemical mutagen Doxorubicin (DX) than both cytoCTD-expressing and control cells (Fig. S3).

The increased sensitivities to IR and DX that result from expressing the nucCTD are consistent with the idea that this fusion protein competes with the endogenous PCTD of elongating RNAPII for binding to PCAPs; an example of nucCTD binding for one PCAP, Set2, is presented in Fig. S2. Elongating RNAPII is thus left with a depleted or disarrayed set of CTD-associating proteins and appears to be defective in mediating an effective DNA damage response.

### Proper CTD Phosphorylation and DNA Repair *via* Homologous Recombination

To further investigate dependence of a normal DNA damage response on proper protein•PCTD interactions, we analyzed strains in which the phosphorylation state of the CTD is altered, namely *ctk1*Δ strains [33]–[36]. As mentioned earlier, it is already known that *ctk1*Δ strains show increased sensitivity to several kinds of physical and chemical DNA damage (see Table S1); this is despite the fact that the expression of DNA damage response genes appears unaltered in *ctk1*Δ strains [37]. Interestingly, while most of these studies used haploid strains, one employing diploid strains [32] reported some unique findings. Westmoreland et al. found, for example, that *ctk1*Δ*/ctk1*Δ diploids were several fold more sensitive to Doxorubicin (DX) than were *ctk1*Δ haploids, and the same diploid-specificity held for a number of other damage-resistance loci. Because one line of explanation for diploid specificity invokes participation of HR-dependent repair events in diploids that cannot occur in haploids [29], [30], we were motivated to investigate potential connections between *CTK1* and HR-dependent DNA repair.

The literature already contains strong suggestions in support of such connections. Strains deleted for genes encoding subunits of CTDK-I (e.g. *ctk1*Δ, *ctk2*Δ and *ctk3*Δ strains) display synthetic lethality (SL) with mutations in well known HR genes (noted in “Supplemental Results and Discussion” of ref [28], for example). When we checked the SGD (Saccharomyces Genome Database, http://www.yeastgenome.org), for all 74 “DNA integrity” (DI) genes subjected to genome-wide SL screens by Pan et al. [28], we found that 38 DI genes displayed synthetic lethality (or synthetic growth defects) with *ctk1*Δ, *ctk2*Δ, and/or *ctk3*Δ (see Table S2) (i.e. over half of the DI genes). Notably, among these 38 genes were 8 genes encoding the major HR proteins. Because SL interactions frequently indicate related but complementary functions [28], [38], the interactions between *ctk*Δ genes and HR plus other DI genes support the concept that CTDK-I enables a function or functions complementary to already-known pathways for maintaining DNA integrity, presumably by generating the hyperphosphorylated CTD to which CAR proteins bind.

### Spontaneous Mitotic Recombination Depends on Ctk1

One system suitable for looking into a role for CTDK-I in HR-dependent repair is that of spontaneous mitotic recombination. In mitotically growing diploid yeast cells, homologous recombination (HR) occurs at a low spontaneous rate [39], and this rate is increased by DNA damaging agents [40], [41]); thus, mitotic recombination reflects the presence and repair of DNA damage. Mitotic recombinational repair occurs principally *via* HR, which can of course repair DSBs; however, in untreated, normally growing cells it is now thought that the lesions provoking spontaneous recombinational repair are largely single strand nicks and gaps [42].

**Figure 2:**
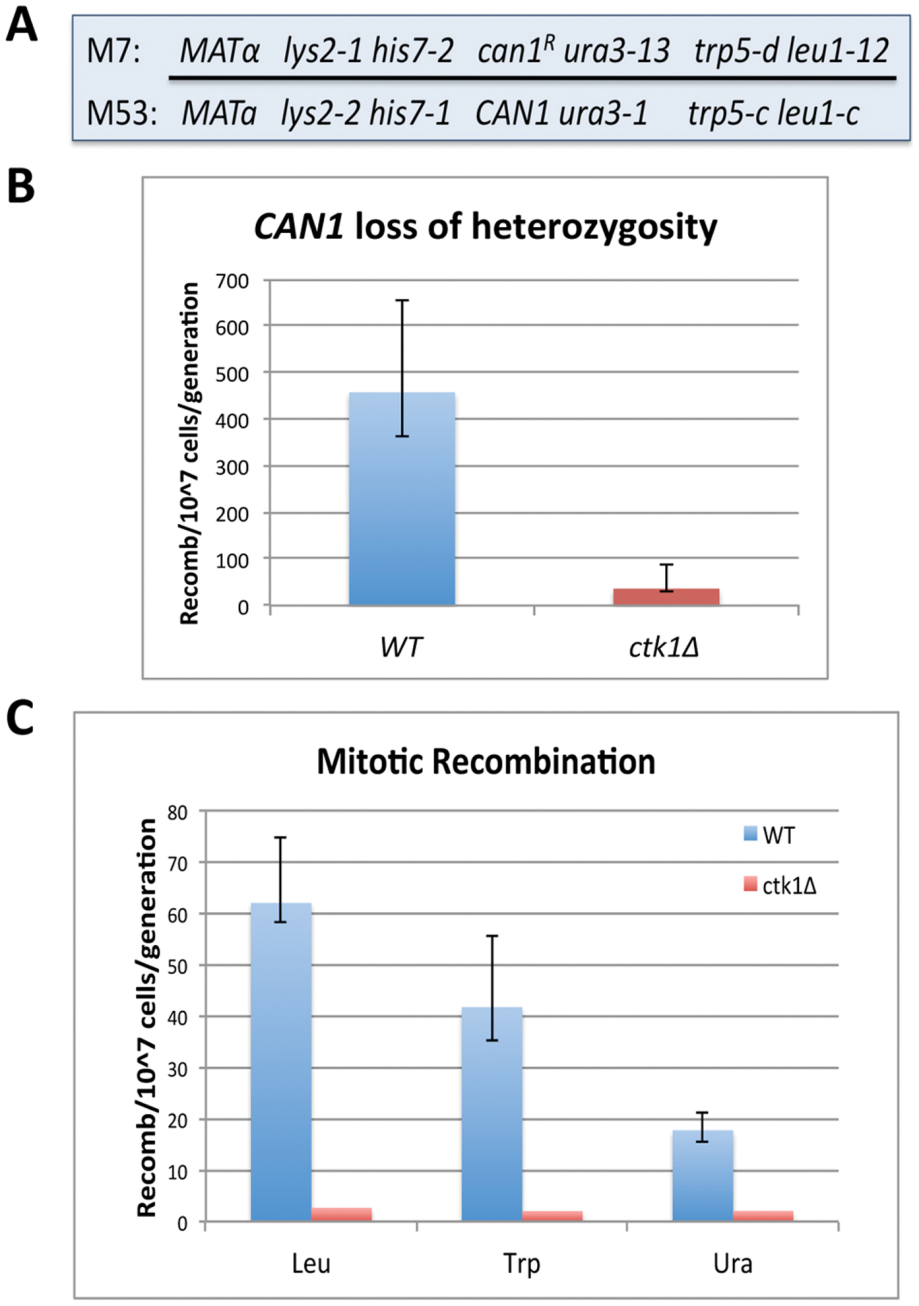
Spontaneous mitotic recombination depends on normal CTD phosphorylation. *(A)* Schematic of alleles in strains M7 and M53, used for generating diploid strains employed in mitotic recombination assays. *(B)* LOH at *CAN1* in *CTK1/CTK1 (WT)* and *ctk1*Δ*/ctk1*Δ strains. Error bars indicate 95% confidence intervals. Following deletion of *CTK1*, strains exhibit a 10-fold decrease in LOH (reflects spontaneous mitotic recombination between centromere and *CAN1* locus). *(C)* Mitotic recombination assays comparing *WT* with *ctk1*Δ*/ctk1*Δ strain. Error bars indicate 95% confidence intervals. Deletion of *CTK1* causes a 5–20 fold decrease in recombination rates at three different heteroalleles.

If a properly phosphorylated CTD is needed for repair of these lesions, interfering with CTDK-I function should affect rates of spontaneous mitotic recombination. To test this expectation, we employed a test strain (M7/M53) containing hetero-alleles at several auxotrophic loci (Fig. 2A) [43] (and Table S5). Because each hetero-allele encodes a defective protein, recombination between hetero-alleles is required to generate a functional gene, expression of which allows growth on selective medium. The test strain is also heterozygous at the *CAN1* locus, carrying a (dominant) canavanine-sensitivity allele (*CAN1*) and a (recessive) canavanine-resistance allele (*can1^R^*). Canavanine-resistance can result from a recombination event anywhere between the *CAN1* locus (located near the end of chromosome V) and the centromere of chromosome V (a distance of ∼120 kb), followed by appropriate chromosome disjunction to yield a progeny cell homozygous for *can1^R^*. Thus the loss of heterozygosity (“LOH”) at *CAN1* can serve as a proxy for recombination within this 120 kb chromosomal segment. In order to check for alterations in spontaneous mitotic recombination rates as a function of CTD phosphorylation, we generated a *ctk1*Δ*/ctk1*Δ (kinase deficient) diploid strain otherwise isogenic to M7/M53, which is kinase proficient (*CTK1*/*CTK1*). It is important to note that RNAPII transcription *per se* and RNAPII genome distribution are largely unchanged by *CTK1* deletion [35]–[37], whereas some pre-mRNA processing events are affected [20], [35], [37]. It is also notable that gene expression comparisons between *CTK1 WT* and *ctk1*Δ strains, as assessed by Affimetrix genome array analysis of mRNAs, do not reveal perturbations for DNA repair or recombination genes in *ctk1*Δ strains [37].

In the *CTK1/CTK1* diploid, we found the rate for loss of heterozygosity at *CAN1* to be ∼450 recombinants per 10^7^ cells per generation (Fig. 2B, “*WT*”). Strikingly, this rate was 10-fold lower in the *ctk1*Δ*/ctk1*Δ strain (Fig. 2B, “*ctk1*Δ”). Similarly, spontaneous mitotic recombination rates at *LEU1, TRP5* and *URA3*, which range from ∼ 20 to 60 recombinants per 10^7^ cells per generation in *WT (CTK1/CTK1)* cells, were reduced 5- to 20-fold in the *ctk1*Δ/ctk1Δ strain (Fig. 2C). A control experiment showed that re-introducing a plasmid-borne *CTK1*-*WT* gene into the *ctk1*Δ*/ctk1*Δ strain brought back higher recombination rates (data not shown). Thus, ∼90% of spontaneous mitotic recombination events in normally growing diploid cells depend on *CTK1*. A reasonable interpretation of these results is that most HR-mediated repair of spontaneous DNA damage in mitotically growing diploids requires a properly-phosphorylated CTD on elongating RNAPII.

### PCTD Binding Ability of Set2 is Needed for its Role in DNA Damage Resistance

**Figure 3:**
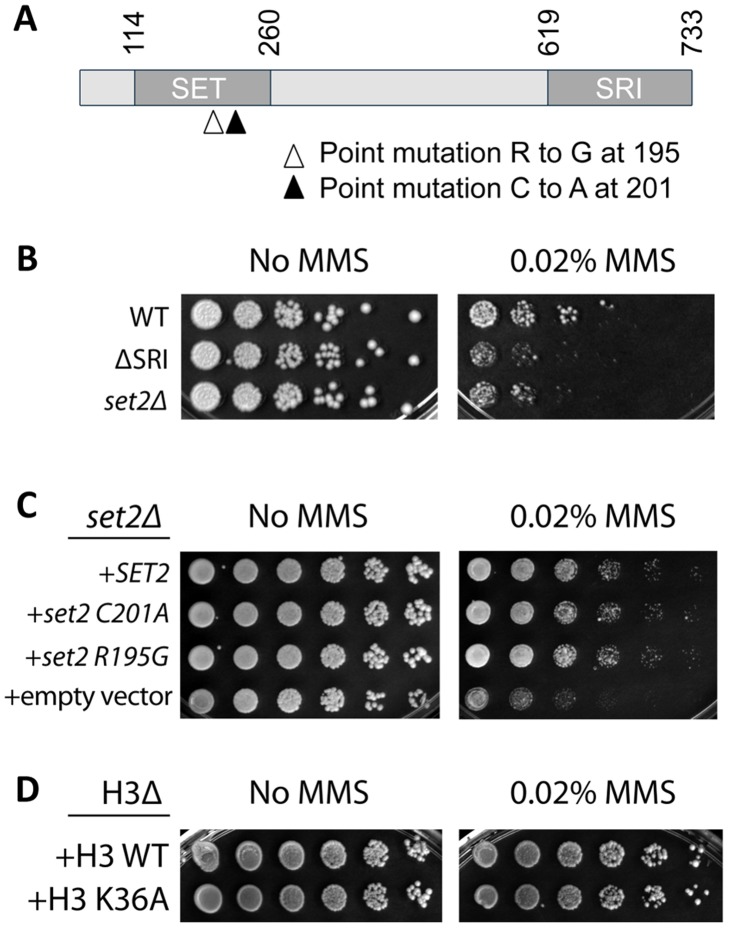
Damage resistance function of Set2 requires phosphoCTD binding but not catalytic activity. *(A)* Primary structure of Set2 showing SET (catalytic) domain and SRI (CTD binding) domain. Position of catalytic point mutations is also illustrated. *(B)* The SRI domain of Set2 is required for damage resistance. Serial dilutions of *SET2 WT*, Δ*SRI* and complete gene deletion (*set2*Δ) strains were spotted on rich (YPD) medium containing either zero or 0.02% MMS, grown for 3 days at 30°C and photographed. “No MMS” results show that very similar numbers of cells were spotted for the three strains and that growth rates are quite similar (size of isolated colonies). All strains were affected by 0.02% MMS, for both survival and growth rate, but relative to WT (“normal” level of resistance) *set2*Δ and Δ*SRI* were less resistant. *(C)* Damage resistance does not depend on histone methyltransferase activity of Set2. Five-fold serial dilutions of *set2*Δ strains covered by various mutants of Set2 were plated with or without 0.02% MMS. Catalytically “dead” point mutants (C201A and R195G) are as resistant to MMS as the WT allele. *(D)* Methylation of H3 K36 is not required for resistance to MMS. Five-fold serial dilutions of strains in which histone genes are deleted from the genome but covered by either a plasmid with WT histones (top row) or a plasmid in which H3 carries a K36A point mutation (bottom row) were plated with and without 0.02% MMS. There is no observable difference in survivability between the WT strain and the mutant strain on MMS.

A likely implication of the results above is that disturbing the PCTD interaction of certain individual PCAPs would result in DNA damage sensitivity. Since we had previously characterized the PCTD-binding properties of the PCAP Set2, a transcription elongation-linked histone methyltransferase Set2 (Fig. 3A) [44], [45], we decided to check whether Set2 plays a role in DNA damage resistance and, if it does, to determine the importance of its PCTD-interacting domain (the SRI [Set2-Rpb1-interacting] domain; Fig. 3A) [44], [46] for that role.

We first compared damage resistance of a *set2*Δ strain with that of a *SET2* WT strain, and we found that deleting the entire *SET2* gene indeed increases sensitivity to several DNA damaging agents, including methylmethanesulfonate (MMS) (Fig. 3B), IR and DX (Fig. S4). We next tested a mutant version of *SET2* from which the SRI domain was deleted (Δ*SRI*; see Fig. 3); the results show that deleting the SRI domain alone leads to damage sensitivity for MMS (Fig. 3B), as well as for DX (Fig. S4) and IR. Notably, deleting the SRI domain increased damage sensitivity at least as much as, if not more than, deleting the whole protein (Fig. 3B, Fig. S4). These results suggest that the Set2-ΔSRI protein fragment (residues 1–619), which is catalytically active but does not associate properly with the RNAPII transcription elongation apparatus [44], displays a dominant negative effect in terms of its role in the DNA damage response. Most importantly, these results demonstrate that the role of Set2 in DNA damage resistance depends on its proper interaction with the PCTD.

### The Enzymatic Activity of Set2 is *not* Required for Damage Resistance

While the Set2 SRI deletion experiment shows that the protein needs to associate properly with the PCTD to perform its role in damage resistance, the experiment does not address whether or not the histone methyl transferase catalytic activity (HMTase) of Set2 is required. It is possible, for example, that damage resistance mediated by Set2 protein might not depend on its enzymatic activity but just on the physical presence of the full-length protein and its proper association with the CTD. We tested this idea by employing catalytically-impaired but full-length versions of Set2, mutants *C201A* and *R195G* [47]. Transforming a diploid *set2*Δ*/set2*Δ strain with a plasmid expressing *WT* Set2 provides damage resistance (Fig. 3C, top row, “+*SET2*”), while transforming with empty vector does not (Fig. 3C, bottom row, “+ empty vector”). Remarkably, transforming with plasmids expressing the full length but catalytically defective proteins restores damage resistance to the *WT* level (Fig. 3C, 2^nd^ and 3^rd^ rows). These results suggest a non-catalytic role for Set2 in the CAR system. As a check on the dispensability of the H3K36 methyltransferase activity of Set2, we tested DNA damage resistance levels of a *SET2/SET2 (WT)* strain expressing only a mutant version of histone H3 in which Set2’s methylation target (K36) was changed to non-methylatable alanine (H3[K36A]). Consistent with the mutant enzyme results, mutant histone H3[K36A] provided the same level of damage resistance as did *WT* H3 (Fig. 3C). These results confirm that H3-K36 histone methyltransferase activity is not required for the role Set2 plays in DNA damage resistance; notwithstanding, the protein must be present and make proper contacts with the PCTD.

### A Subset of PCAPs Required for IR resistance: the CAR Proteins

**Figure 4:**
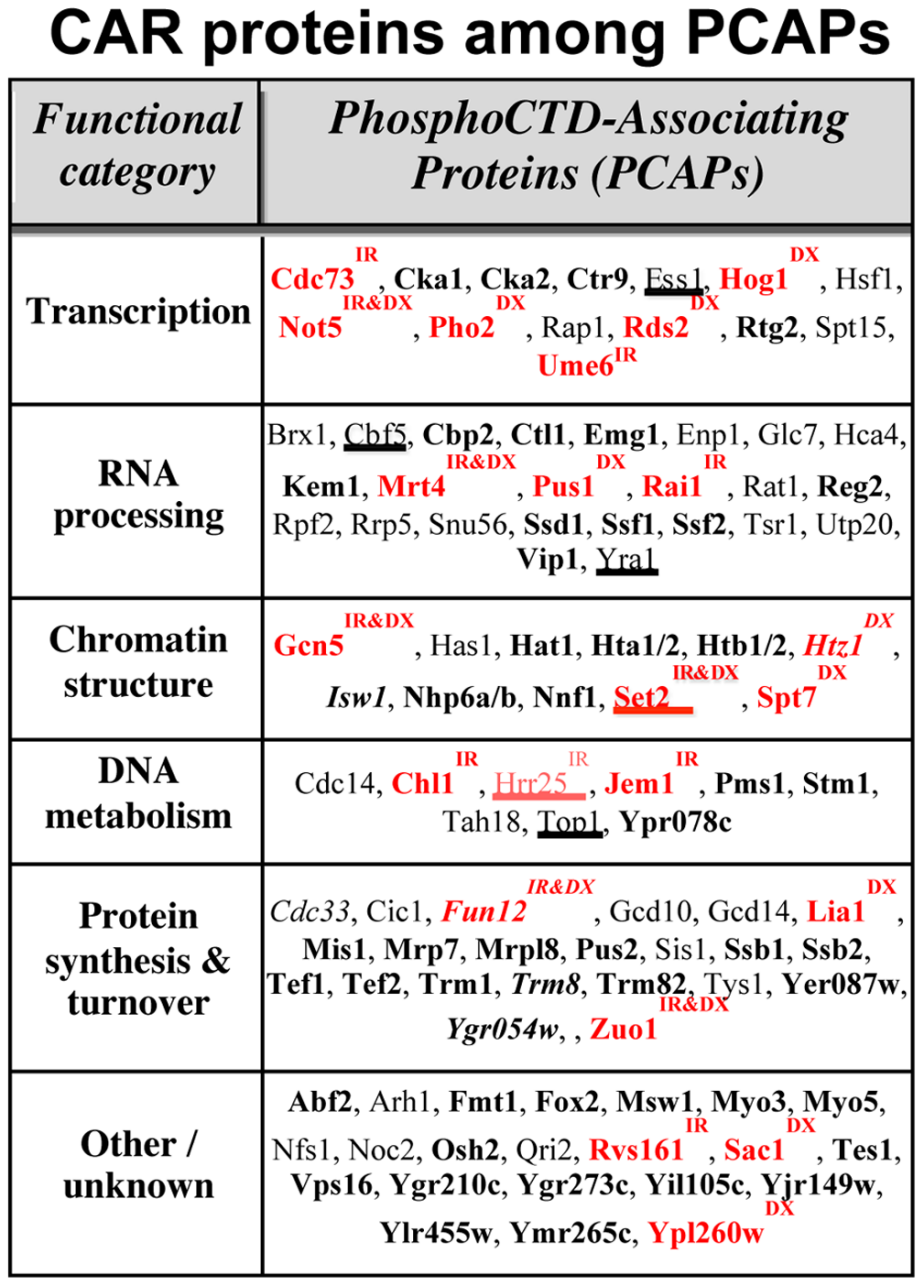
PhosphoCTD-associating proteins (PCAPs) were identified by Phatnani et al. [**20**] and assigned to likely functional categories. Proteins in red (CAR proteins) are products of genes identified by Bennett and colleagues as required for normal resistance to ionizing radiation (IR) (23,24) or doxorubicin (DX) (26). Bold = non-essential; light = essential; underlined = binds directly to PCTD; *italics = does not bind directly to PCTD*.

Given the results above, it appears that Set2 belongs to a subset of PCAPs, required for DNA damage resistance, which we will refer to as CAR (CTD-associating damage response) proteins. In order to determine how many other PCAPs might be CAR proteins, we made use of some published data and came up with a very surprising finding. Previously, we used a stringent affinity-isolation approach to purify *S. cerevisiae* proteins that associate with CTD peptides phosphorylated in a pattern generated by CTDK-I (Ser2,5P heptad repeats); we subsequently employed mass spectrometry to identify approximately 100 of these PCAPs [20]. Included among the 100 PCAPs is a wide variety of proteins, many of which had not been previously connected with transcription (Fig. 4). When we compared our list of PCAPs with a list of damage resistance genes identified by Bennett and colleagues through screening the yeast diploid deletion collection [29], [30], [32], we obtained an amazing result: overlap of the two lists was 5-fold higher than predicted by random chance. Figure 4 illustrates that, among 72 non-essential genes encoding PCAPs, 12 are required for ionizing radiation (IR) resistance and an overlapping 15 for Doxorubicin (DX) resistance (note that Bennett and colleagues, using double deletion strains, could not analyze “essential” genes, and proteins encoded by such genes are indicated in Fig. 4 by light font). We emphasize that the overlap between the PCAP and IR “damage-resistance” data sets is much higher than expected by chance, and that chi squared analysis of the overlap between the PCAPs and IR resistance genes gives a P value of <0.0001 (see Methods). The overlap between the DX resistance group and PCAPs is likewise highly significant.

The PCAPs that are also needed for DNA damage resistance comprise a previously-unrecognized set of transcription elongation-linked DNA damage response proteins; they fall into the category we defined above as “CAR” proteins. One intriguing aspect of the CAR proteins is the extreme diversity of their known functions; they do not represent just the “DNA Metabolism” category of Fig. 4. but populate all of the known functional groups represented by PCTD-associating proteins. It appears that the only two features common to all CAR proteins are that they associate with the PCTD and that they are needed for resistance to DNA damage.

## Discussion

Our data reveal that the hyperphosphorylated CTD of elongating RNAPII organizes a DNA damage response system that involves a significant subset of phosphoCTD-associating proteins, the CAR (CTD-associated damage response) proteins. As signaled by reduced damage resistance, the system can be disrupted by expressing a decoy CTD fusion protein that, when in the nucleus, competes for PCAP binding with the *bona fide* CTD. Moreover, as assessed by measuring rates of spontaneous mitotic recombination, activity of the CAR system in HR-mediated repair is diminished by improper CTD phosphorylation, as in *ctk1*Δ*/ctk1*Δ strains. Finally, whereas numerous PCAPs comprise the CAR system, deleting the PCTD binding domain of just one CAR protein leads to reduced DNA damage resistance. Remarkably, in at least one case, a CAR protein (Set2) with its PCTD binding domain intact supports normal damage resistance levels even when its catalytic activity is crippled.

While PCAPs were already known to be involved in a wide range of cellular processes that include transcription, RNA processing, nuclear RNA export, modulation of chromatin structure, and various transactions of DNA, it was nevertheless surprising to find that deletion of about one in five PCAP genes results in cells that do not respond normally to DNA damage (discovered using the diploid deletion strain collection, *cf*. [29], [30]). For example, deleting *RAI1*, encoding a PCAP involved in transcription termination [48], leads to damage sensitivity (Fig. 4). Similarly, deleting *SAC1*, encoding a PCAP with inositol polyphosphatase activity, likewise leads to damage sensitivity. The data in Fig. 4 indicate that 21 of the 100 PCAPs defined by Phatnani et al. [20] are CAR proteins. In addition to our data, other reports in the literature are consistent with the existence of PCTD/PCAP-mediated responses to DNA damage (also see Introduction). For example, the helicase Sen1 binds the PCTD [49], [50] and is required for preventing transcription-associated genome instability; in this role it appears to function by reducing R loop formation during RNAPII transcription [8]. Also, a recent paper implicates Rtt103, another PCTD-binding protein involved in transcription termination [48], in DNA damage resistance and repair [9]. Altogether, new and existing data strongly support the notion that a major function of the PCTD on elongating RNAPII is to maintain the integrity of the genome.

Several kinds of evidence support the idea that CAR system activity depends on physical association of CAR proteins with the PCTD of elongating RNAPII. First, interfering physically with global PCAP**–**PCTD interactions in the nucleus impairs CAR system function (Fig. 1). Second, for at least one CAR protein (Set2), debilitating its PCTD binding capacity, while leaving its enzymatic activity intact, compromises CAR activity. Third, and in stark contrast to the preceding result, mutating the catalytic activity of Set2 in the context of the full-length protein does *not* affect CAR system function (Fig. 3). Taken together, the results with Set2 make a strong argument that its observed role in DNA damage resistance depends on the proper physical association of Set2 with elongating RNAPII. Finally, an aberrantly phosphorylated CTD, as found in *ctk1*Δ strains, fails to support CAR system function; both resistance to DNA damaging agents (*cf.* Table S1) and rates of HR-based spontaneous mitotic recombination (Fig. 2) are affected. Together, these results bear out the importance of correct physical association of CAR proteins with a properly phosphorylated CTD as found on elongating RNAPII.

**Figure 5:**
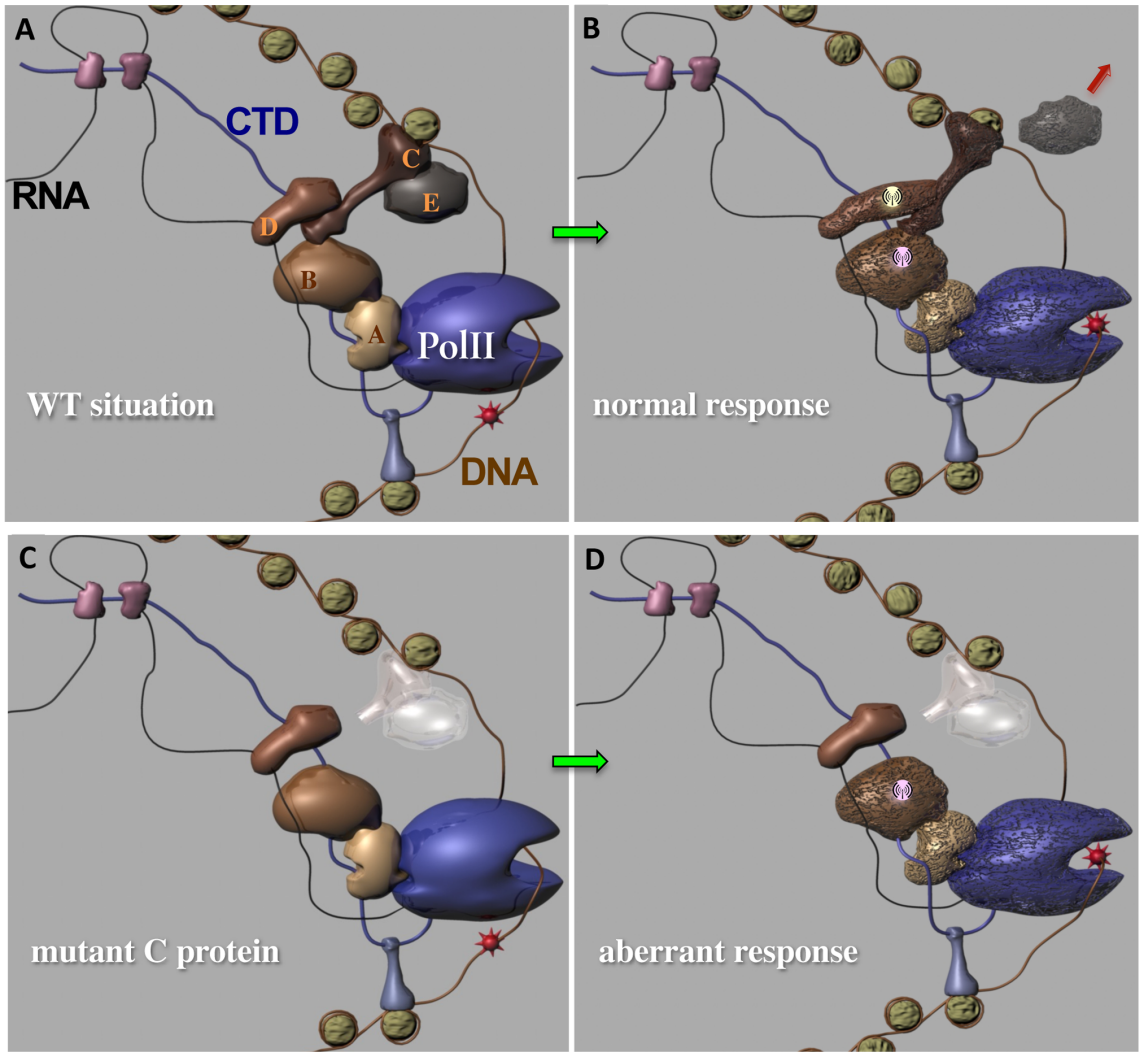
Speculative model of the CAR system. *(* ***A*** *)* CAR proteins associate with elongating RNA Polymerase II through interactions with the phosphoCTD. Elongating Pol II (dark blue) is depicted with 3 classes of PCTD associating proteins (shades of pink, brown, and blue, respectively) bound either directly or indirectly to the normally-phosphorylated CTD (dark blue line). We speculate that some CAR proteins form complexes with particular functions; here, for example, we propose that A, B, C, D and E comprise a CAR complex that functions as a damage-responsive module; note how it is coupled to the globular catalytic core of PolII. The elongating catalytic core of Pol II will soon encounter a translocation-blocking DNA lesion (red star on orange DNA). *(* ***B*** *)* CAR proteins respond to damage that blocks elongation. The catalytic core of Pol II has collided with the lesion; translocation is blocked. Changes ensue, signaling that polymerase is blocked and ultimately leading to repair. Possible changes include: 1) alterations in conformation, depicted by shape and surface changes (of core and proteins coupled to it, such as A and B, C, D and E); 2) dissociations and signaling (protein E); 3) changes in activity (not indicated); 4) covalent modifications (beacons on B & D) that signal and/or recruit other components. The combined changes comprise the normal damage response, that leads to repair and normal “damage resistance.” *(* ***C*** *)* An abnormal CAR complex results in aberrant damage response. Here, CAR protein C is truncated, missing its PCTD interacting domain (e.g. SRI domain of Set2). C and E are shown in ghostly white, because in reality they would not properly associate with the elongation complex. Note also that D is no longer coupled to the catalytic core. *(* ***D*** *)* A disrupted CAR system leads to reduced damage resistance. When damage blocks the movement of PolII, changes are induced, but their extent is diminished due to absence of PCTD-binding by mutated protein C. Signaling is reduced (signaling from D and E does not occur). Because only a partial damage response is generated, a reduction in damage resistance is observed. We expect that the specific defects underlying a reduced damage response will differ from case to case, depending on which CAR protein is defective and on the nature of the DNA damage.

Our hypothetical model for a CTD-associated DNA damage response system is presented in Figure 5. During the elongation phase of transcription, RNAPII (PolII) is accompanied by a complement of PCTD-associated proteins (only a subset is depicted in Fig. 5A). Note that some PCAPs also bind the transcript and some interact with chromatin; in addition, some bind the PCTD directly and some bind it indirectly. As presented here, PCAP A interacts with both the PCTD and the globular core of the polymerase. When a transcription-blocking DNA lesion (red star) impacts the enzyme (Fig. 5B), changes ensue in the globular core (indicated by altered shape and surface) that are communicated to a subset of the PCAPs (e.g. module A, B, C, D, E), potentially through protein protein contacts as depicted here (Fig. 5B). Affected PCAPs participate in signaling that PolII is blocked, potentially utilizing a number of mechanisms such as dissociation (E) or modification (of surface or catalytic activity) (B, D). In wild type cells these events together comprise the normal damage signaling response (Fig. 5B).

In a CAR protein mutant cell (Fig. 5C & D) the signaling chain would be broken, for example, if PCAP C were missing its PCTD-binding domain (Fig. 5C). In this case, several important protein•protein contacts would be absent throughout this phase of elongation, and a number of PCAPS/CAR proteins would be improperly bound to the PCTD, if bound at all (e.g. PCAPs C & E). Consequently only a fraction of the normal signaling events would occur when PolII is blocked by the DNA lesion (Fig. 5D). The net result will be an aberrant damage response, as revealed, for example, by increased damage sensitivity.

It seems likely that one downstream consequence of CAR system signaling will be activation of a damage checkpoint (e.g. [11]), and another will be ubiquitinylation and degradation of PolII (e.g. [4]). Especially if the damage is to be repaired through HR mediated events, “clearing” the huge transcription elongation complex off the DNA might be necessary, and degrading the PolII would be part of this process. It has already been speculated in the literature that if transcription coupled nucleotide excision repair (TC-NER; mediated by Rad26 [mammalian CSB]) fails to remove a transcription-obstructing lesion, “… RNAPII is polyubiquitylated and eventually degraded - as a last resort. … RNAPII degradation would then free the DNA lesion to be removed by other means, for example by … DNA recombination…” [4]. These ideas fit nicely with our CAR system model, except, rather than being just a “last resort” mechanism, we suggest that the CAR system in fact evolved to deal with the numerous types of transcription-blocking DNA lesions that are refractory to TC-NER.

Our findings raise many new questions. For example, is the CAR system a single, monolithic entity or is it composed of smaller modules? In view of the very different DNA insults to which the cell responds in a PCTD/PCAP-dependent manner, it seems likely that distinct responses might be mounted by different (groups of) CAR proteins. Consistent with this idea, we found that the 38 DNA integrity (DI) genes showing synthetic lethality with *CTK* deletions (Table S2) displayed different patterns of synthetic lethality with CAR protein genes (Table S3). For example, five *CAR* genes (*CDC73, RVS161, SAC1, SET2* and *UME6*) share 3 or more SL interactions with a group of DI genes that were placed in closely related modules by Pan et al. [28] (*ARD1, ASF1, BRE1, CCR4, LGE1, MDM39, NAT1, RAD27, RAD6, RMD7*). In contrast, *CHL1* is very different, displaying SL interactions with four DI genes that are not SL with any other CAR protein genes. If we assume that shared SL interactions indicate functional relatedness [28], [38], then *CDC73, RVS161, SAC1, SET2* and *UME6* are likely to be involved in the same or similar functions; they could even be considered a functional module. Along similar lines, *CHL1* may be the singele member of another module. It will be extremely interesting to see what future experiments reveal about the actual functional and physical interactions among these important macromolecules.

Most components of the CAR system are evolutionarily conserved from yeast to human beings (Table S4), leading us to propose that the CAR system is present and operational in human cells. Moreover, since mutations in human DNA damage response genes often reduce genome stability and are frequently oncogenic, we predict that mutations in human CAR system genes underlie certain types of cancer. Already fulfilling this prediction are results from several recent studies of human cancers. For example, a large study of high-grade serous ovarian cancer reveals human CDK12, the counterpart of yeast Ctk1 [51], to be a tumor suppressor [52]; CDK12 is also significantly mutated in prostate cancer [53] and in several other cancers (http://www.cbioportal.org/public-portal/). We suggest that cancer-causing mutations in CDK12 debilitate the human CAR system and that this contributes to genome instability and cancer. As well, we point out that mammalian orthologs of other yeast CAR protein genes are also linked to cancer. Notably, the Set2 counterpart, SETD2 (Table S4) shows high mutation rates in several cancers, and in a number of cases the SRI domain is altered (<http://www.cbioportal.org>). Another example is human CDC73 (also known as HRPT2; encodes “parafibromin”), in which mutations lead to parathyroid cancer [54]. One more example is DNAJC2, the human counterpart of Zuo1, that is significantly mutated in a number of cancers (<http://www.cbioportal.org>). We hypothesize that a feature common to these seemingly-unrelated tumor suppressors is their participation in the CAR system. To conclude, we point out that through detailed “discovery” work in a simple model organism we uncovered a fundamental, conserved DNA damage response system. A better understanding of the CAR system, obtained through future studies in both simple and complex organisms, should eventually provide opportunities for developing novel methods and medicines to treat cancers and bring about improvements in human health.

## Methods

### Strains and Plasmids

Yeast strains (Table S5) were diploids from the BY4743 background, unless otherwise noted, and extant deletion strains were obtained from the yeast deletion collection [55]. DNA damage experiments and survival tests were generally carried out as described [29], [30], with doses and amounts given in the figures and legends. IR experiments were done in BY4743.

The nucCTD and cytoCTD plasmids were generated from pTCM-RA and pTCM-RR [56] by restriction digestion of a ßgalactosidase-CTD fusion construct [57] at the Sst1 site internal to the LacZ gene (leaves a ⅔-length ß-Galactosidase) and an EcoRI site downstream of the CTD sequence, and ligation into the corresponding sites of the pTCM plasmid (courtesy of J.M. Lee). To construct the nucßGal plasmid we used Pfu DNA Polymerase (Invitrogen) to amplify the portion of the *LacZ* gene used in the nucCTD plasmid from pTCM-RA. The resulting PCR product was then purified using a PCR clean-up kit (Qiagen) and incubated for 10 minutes at 72 degrees with taq polymerase and dNTPs to add adenine overhangs on the 3′ ends of the PCR product. We then used the Gateway system (Invitrogen) and TOPO cloning to clone this product into an ENTRY vector. The resulting ENTRY vector was used in an LR recombination reaction to transfer the nucßGal sequence to pYES DEST52. All cloning reactions were performed according to the manufacturer’s instructions (Invitrogen).

All *SET2* mutant strains were diploids of BY4743 background. The *WT* and *set2*Δ were obtained from the yeast deletion collection. The Δ*SRI* strain was constructed by mating a BY4742 *set2*Δ*SRI::KanMX* strain with a BY4741 *set2*Δ strain. Both haploids were a gift from Brian Strahl. See table for full genotype.

The histone mutant strains were constructed using LRY1443 and LRY1444. These strains both have histones H3 and H4 knocked out of the genome and they are covered with the pDM9 plasmid (which contains *HHT1* and *HHF1* as well as a *URA3* marker). This plasmid was then replaced using a standard plasmid shuffle assay with another plasmid containing *TRP1*, *HHT1*, and either *HHF1* (+*WT H3* strain) or *hhf1 K36A* (+*H3 K36A* strain). The haploid strains were then mated and mated structures were picked using a dissection microscope to yield diploid strains. Strains and plasmids were a gift from Laura Rusche. Plasmids are originally from the lab of Fred Winston [58].

Mitotic Recombination: The “*WT*” haploid yeast strains M7 and M53 were obtained from Kevin Lewis at the University of Texas, but were originally constructed by Robert Malone [43]. To construct homozygous diploids deleted for CAR protein genes, the *CAR* gene was first deleted in both M7 (*MATα*) and M53 (*MAT* ***a***) haploids, and these were then mated to produce the ***a***/*α car*Δ*/car*Δ strain. Deletions were checked via PCR and diploidy was checked by mating and auxotrophy tests.

### IR Sensitivity Assay

Irradiations were preformed as described in Bennett et al. [29]. Briefly, for dilution plating assays, plasmid-containing cells were grown for two days in a 96 well dish in CM+gal-ura. Five-fold serial dilutions were made, plated to CM+gal-ura plates, and irradiated. For survival curves, cells were pre-grown for 24 hours in liquid CM+gal-ura, diluted in water, and irradiated. Irradiated cells were then plated to CM+gal-ura plates and colony counts were compared to plates from the same dilution where the cells had not been irradiated.

### MMS Sensitivity Assays

Cells were grown in a 96 well plate in either YPD (for genomic mutants and histone mutants) or complete medium lacking uracil (CM-ura) for 2 days. Stationary phase cells were then diluted in sterile H_2_O by 5-fold serial dilutions and plated to YPD or CM-ura plates that either did or did not contain methyl methanesulfonate (MMS). For plates containing MMS (Sigma, 129925), 0.02% MMS was added to warm agar media immediately before plates were poured.

### Mitotic Recombination Assays

Mitotic Recombination assays were performed based on the protocol in Malone and Hoekstra [59]. Two days prior to starting the liquid cultures (at least 12 independent cultures per genotype), diploid strains were streaked to YP4%D (YPD with 4% dextrose) plates and colonies were allowed to form. For each culture, a colony was resuspended in sterile H_2_O and counted on a hemocytometer. A small number of cells (about 100 cells/mL) was added to liquid YPD and incubated with shaking at 30°C to a final cell density of approximately 1×10^7^ cells/mL. Various dilutions were plated to YPD; YPD containing cyclohexamide; drop out media lacking leucine, uracil, or histidine; and drop out media lacking arginine but containing canavanine. Plates were incubated at 30°C for several days and then scored.

### Mutation Rate Calculations

We performed fluctuation analysis using the Lea-Coulson Method of the Median [60] using the FALCOR website [61].

## Supporting Information

Figure S1 **CTD fusion protein constructs, expression, phosphorylation and localization. A.** Plasmids for expression of CTD fusion proteins. NLS, Nuclear Localization Signal; ßGal, N-terminal 2/3 of ß-Galactosidase. **B.** Western Blots of extracts with uninduced (U) or induced (I) CTD fusion proteins. Antibodies against Ser5 phosphorylated (anti 5P), Ser2 phosphorylated (anti 2P), hyper-phosphorylated CTD (anti 2,5P), ß-Galactosidase (anti ßGal), and a loading control (anti pgk1), show that the fusion proteins are phosphorylated. **C.** Immunoflourescense of strains expressing the fusion proteins. Comparing nuclear staining (DAPI) with fusion protein expression (anti-ßGal) shows that the fusion proteins are properly localized.
(PDF)

Figure S2 **Pull-down of Set2 by CTD fusion proteins.** Fusion proteins were expressed in WT yeast cells and pulled down by immunoprecipitation (IP) with an anti ß-Galactosidase antibody. Co-IP of Set2 is illustrated via western blot using an antibody against Set2. Onput (OP) and Flow Through (FT) show Set2 is present in the extract. IP from extract in which nucCTD is expressed shows that Set2 is pulled down by nucCTD fusion protein; however, Set2 is not pulled down by nucßgal fusion protein (lacking a CTD).
(PDF)

Figure S3 **Interfering with binding of PCAPs to the CTD of elongating RNAPII leads to Doxorubicin (DX) sensitivity.** NucCTD-, cytoCTD- and empty vector-carrying strains (as in Fig. 1) were spotted in 5-fold serial dilutions on plates containing glucose (Non-induced) or galactose (Induced) and either 0 or 25 µg/ml DX, grown at 30°C for 3 days and photographed. Note that while the nucCTD strain grows slower than the others under inducing conditions, the presence of DX accentuates the difference between it and the other two strains (for nucCTD, colonies are present in all six dilution spots in the absence of DX but are not present in the rightmost two spots in the presence of DX).
(PDF)

Figure S4 **The SRI domain of Set2 is required for damage resistance.** Serial dilutions of *SET2 WT*, Δ*SRI* and complete gene deletion (*set2*Δ) strains were spotted on rich (YPD) medium containing either zero or 25 µg/ml DX, grown for 3 days at 30°C and photographed. “No Doxorubicin” results show that very similar numbers of cells were spotted for the three strains and that growth rates are quite similar (size of isolated colonies).
(PDF)

Table S1 **ctk1Δ strains are sensitive to DNA damaging agents.**
(PDF)

Table S2 **CTK1,2,3 show synthetic lethal relationships with HR genes and "DNA-Integrity" genes.**
(PDF)

Table S3 (PDF)

Table S4 **Yeast CAR genes, recognized functions, and human orthologs.**
(PDF)

Table S5 **Yeast strains.**
(PDF)

Text S1 **METHODS for Supporting Information.** This file contains descriptions of methods for (1) Localization of fusion proteins, (2) Antibodies and Western Blotting, and (3) Immunoprecipitations.
(DOCX)

## References

[1] Lindsey-Boltz LA, Sancar A (2007) RNA polymerase: the most specific damage recognition protein in cellular responses to DNA damage? Proc Natl Acad Sci USA 104: 13213–13214 doi:10.1073/pnas.0706316104. 17684092 10.1073/pnas.0706316104 PMC1948916

[2] Djebali S, Davis CA, Merkel A, Dobin A, Lassmann T, et al (2012) Landscape of transcription in human cells. Nature 489: 101–108 doi:10.1038/nature11233. 22955620 10.1038/nature11233 PMC3684276

[3] Hanawalt PC, Spivak G (2008) Transcription-coupled DNA repair: two decades of progress and surprises. Nat Rev Mol Cell Biol 9: 958–970 doi:10.1038/nrm2549. 19023283 10.1038/nrm2549

[4] Svejstrup JQ (2010) The interface between transcription and mechanisms maintaining genome integrity. Trends Biochem Sci 35: 333–338 doi:10.1016/j.tibs.2010.02.001. 20194025 10.1016/j.tibs.2010.02.001

[5] Sordet O, Nakamura AJ, Redon CE, Pommier Y (2010) DNA double-strand breaks and ATM activation by transcription-blocking DNA lesions. Cell Cycle 9: 274–278. 20023421 10.4161/cc.9.2.10506 PMC7289056

[6] Pankotai T, Bonhomme C, Chen D, Soutoglou E (2012) DNAPKcs-dependent arrest of RNA polymerase II transcription in the presence of DNA breaks. Nat Struct Mol Biol. doi:10.1038/nsmb.2224. 10.1038/nsmb.2224 22343725

[7] Gandhi M, Evdokimova VN, T Cuenco K, Nikiforova MN, Kelly LM, et al (2012) Homologous chromosomes make contact at the sites of double-strand breaks in genes in somatic G0/G1-phase human cells. PNAS 109: 9454–9459 doi:10.1073/pnas.1205759109. 22645362 10.1073/pnas.1205759109 PMC3386068

[8] Mischo HE, Gómez-González B, Grzechnik P, Rondón AG, Wei W, et al (2011) Yeast Sen1 helicase protects the genome from transcription-associated instability. Mol Cell 41: 21–32 doi:10.1016/j.molcel.2010.12.007. 21211720 10.1016/j.molcel.2010.12.007 PMC3314950

[9] Srividya I, Tirupataiah S, Mishra K (2012) Yeast Transcription Termination Factor Rtt103 Functions in DNA Damage Response. PLoS ONE 7: e31288 doi:10.1371/journal.pone.0031288.t001. 22355353 10.1371/journal.pone.0031288 PMC3280293

[10] Anindya R, Aygün O, Svejstrup JQ (2007) Damage-induced ubiquitylation of human RNA polymerase II by the ubiquitin ligase Nedd4, but not Cockayne syndrome proteins or BRCA1. Mol Cell 28: 386–397 doi:10.1016/j.molcel.2007.10.008. 17996703 10.1016/j.molcel.2007.10.008

[11] Derheimer FA, O'Hagan HM, Krueger HM, Hanasoge S, Paulsen MT, et al (2007) RPA and ATR link transcriptional stress to p53. Proc Natl Acad Sci USA 104: 12778–12783 doi:10.1073/pnas.0705317104. 17616578 10.1073/pnas.0705317104 PMC1937543

[12] Harreman M, Taschner M, Sigurdsson S, Anindya R, Reid J, et al. (2009) Distinct ubiquitin ligases act sequentially for RNA polymerase II polyubiquitylation. Proc Natl Acad Sci USA. doi:10.1073/pnas.0907052106. 10.1073/pnas.0907052106 PMC2778569 19920177

[13] Phatnani HP, Greenleaf AL (2006) Phosphorylation and functions of the RNA polymerase II CTD. Genes Dev 20: 2922–2936 doi:10.1101/gad.1477006. 17079683 10.1101/gad.1477006

[14] Egloff S, Murphy S (2008) Cracking the RNA polymerase II CTD code. Trends Genet 24: 280–288 doi:10.1016/j.tig.2008.03.008. 18457900 10.1016/j.tig.2008.03.008

[15] Buratowski S (2009) Progression through the RNA polymerase II CTD cycle. Mol Cell 36: 541–546 doi:10.1016/j.molcel.2009.10.019. 19941815 10.1016/j.molcel.2009.10.019 PMC3232742

[16] Bartkowiak B, MacKellar AL, Greenleaf AL (2011) Updating the CTD Story: From Tail to Epic. Genet Res Int 2011: 623718. Available:http://eutils.ncbi.nlm.nih.gov/entrez/eutils/elink.fcgi?dbfrom=pubmed&id=22567360&retmode=ref&cmd=prlinks. 10.4061/2011/623718 PMC3335468 22567360

[17] Wong JM, Ingles CJ (2001) A compromised yeast RNA polymerase II enhances UV sensitivity in the absence of global genome nucleotide excision repair. Mol Gen Genet 264: 842–851. Available:http://www.ncbi.nlm.nih.gov/sites/entrez?Db=pubmed&Cmd=Retrieve&list_uids=11254132&dopt=abstractplus. 10.1007/s004380000374 11254132

[18] Morris DP, Phatnani HP, Greenleaf AL (1999) Phospho-carboxyl-terminal domain binding and the role of a prolyl isomerase in pre-mRNA 3'-End formation. J Biol Chem 274: 31583–31587. 10531363 10.1074/jbc.274.44.31583

[19] Ho Y, Mason S, Kobayashi R, Hoekstra M, Andrews B (1997) Role of the casein kinase I isoform, Hrr25, and the cell cycle-regulatory transcription factor, SBF, in the transcriptional response to DNA damage in Saccharomyces cerevisiae. Proc Natl Acad Sci USA 94: 581–586. 9012827 10.1073/pnas.94.2.581 PMC19556

[20] Phatnani HP, Jones JC, Greenleaf AL (2004) Expanding the functional repertoire of CTD kinase I and RNA polymerase II: novel phosphoCTD-associating proteins in the yeast proteome. Biochemistry 43: 15702–15719 doi:10.1021/bi048364h. 15595826 10.1021/bi048364h PMC2879061

[21] Jeong S-J, Kim H-J, Yang Y-J, Seol J-H, Jung B-Y, et al (2005) Role of RNA polymerase II carboxy terminal domain phosphorylation in DNA damage response. J Microbiol 43: 516–522. 16410768

[22] Carty SM, Greenleaf AL (2002) Hyperphosphorylated C-terminal repeat domain-associating proteins in the nuclear proteome link transcription to DNA/chromatin modification and RNA processing. Mol Cell Proteomics 1: 598–610. 12376575 10.1074/mcp.m200029-mcp200

[23] Bouchard VJ, Rouleau M, Poirier GG (2003) PARP-1, a determinant of cell survival in response to DNA damage. Experimental hematology 31: 446–454. Available:http://www.sciencedirect.com/science?_ob=ArticleURL&_udi=B6VP8-48WFCC2-3&_user=38557&_coverDate=06%2F30%2F2003&_rdoc=1&_fmt=high&_orig=search&_origin=search&_sort=d&_docanchor=&view=c&_acct=C000004358&_version=1&_urlVersion=0&_userid=38557&md5=48e1bf470f5ce10574ed738df3faeb68&searchtype=a. 10.1016/s0301-472x(03)00083-3 12829019

[24] Dantzer F, Amé J-C, Schreiber V, Nakamura J, Ménissier-de Murcia J, et al. (2006) Poly(ADP-ribose) polymerase-1 activation during DNA damage and repair. Meth Enzymol 409: 493–510. Available:http://www.sciencedirect.com/science?_ob=ArticleURL&_udi=B7CV2-4K77K52-11&_user=38557&_coverDate=12%2F31%2F2006&_rdoc=1&_fmt=high&_orig=search&_origin=search&_sort=d&_docanchor=&view=c&_acct=C000004358&_version=1&_urlVersion=0&_userid=38557&md5=ef035212982b5e757cca86e4ead7840e&searchtype=a. 10.1016/S0076-6879(05)09029-4 16793420

[25] Kanagaraj R, Huehn D, Mackellar A, Menigatti M, Zheng L, et al (2010) RECQ5 helicase associates with the C-terminal repeat domain of RNA polymerase II during productive elongation phase of transcription. Nucleic Acids Res 38: 8131–8140 doi:10.1093/nar/gkq697. 20705653 10.1093/nar/gkq697 PMC3001069

[26] Wu J, Phatnani HP, Hsieh T-S, Greenleaf AL (2010) The phosphoCTD-interacting domain of Topoisomerase I. Biochem Biophys Res Commun. 397: 117–119 doi:10.1016/j.bbrc.2010.05.081. 10.1016/j.bbrc.2010.05.081 PMC2900466 20493173

[27] Li X, Manley JL (2005) Inactivation of the SR protein splicing factor ASF/SF2 results in genomic instability. CELL 122: 365–378 doi:10.1016/j.cell.2005.06.008. 16096057 10.1016/j.cell.2005.06.008

[28] Pan X, Ye P, Yuan DS, Wang X, Bader JS, et al (2006) A DNA Integrity Network in the Yeast Saccharomyces cerevisiae. CELL 124: 1069–1081 doi:10.1016/j.cell.2005.12.036. 16487579 10.1016/j.cell.2005.12.036

[29] Bennett CB, Lewis LK, Karthikeyan G, Lobachev KS, Jin YH, et al (2001) Genes required for ionizing radiation resistance in yeast. Nat Genet 29: 426–434 doi:10.1038/ng778. 11726929 10.1038/ng778

[30] Westmoreland TJ, Marks JR, Olson JA, Thompson EM, Resnick MA, et al (2004) Cell cycle progression in G1 and S phases is CCR4 dependent following ionizing radiation or replication stress in Saccharomyces cerevisiae. Eukaryotic Cell 3: 430–446. 15075273 10.1128/EC.3.2.430-446.2004 PMC387653

[31] Game JC, Birrell GW, Brown JA, Shibata T, Baccari C, et al. (2003) Use of a genome-wide approach to identify new genes that control resistance of Saccharomyces cerevisiae to ionizing radiation. Radiation research 160: 14–24. Available:http://eutils.ncbi.nlm.nih.gov/entrez/eutils/elink.fcgi?dbfrom=pubmed&id=12816519&retmode=ref&cmd=prlinks. 10.1667/rr3019 12816519

[32] Westmoreland TJ, Wickramasekara SM, Guo AY, Selim AL, Winsor TS, et al (2009) Comparative genome-wide screening identifies a conserved doxorubicin repair network that is diploid specific in Saccharomyces cerevisiae. PLoS ONE 4: e5830 doi:10.1371/journal.pone.0005830. 19503795 10.1371/journal.pone.0005830 PMC2688081

[33] Lee J-M, Greenleaf AL (1991) CTD kinase large subunit is encoded by CTK1, a gene required for normal growth of Saccharomyces cerevisiae. Gene Expression 1: 149–167. 1820212 PMC5952209

[34] Cho E-J, Kobor MS, Kim M, Greenblatt J, Buratowski S (2001) Opposing effects of Ctk1 kinase and Fcp1 phosphatase at Ser 2 of the RNA polymerase II C-terminal domain. Genes Dev 15: 3319–3329 doi:10.1101/gad.935901. 11751637 10.1101/gad.935901 PMC312848

[35] Ahn SH, Kim M, Buratowski S (2004) Phosphorylation of serine 2 within the RNA polymerase II C-terminal domain couples transcription and 3' end processing. Mol Cell 13: 67–76. 14731395 10.1016/s1097-2765(03)00492-1

[36] Qiu H, Hu C, Hinnebusch AG (2009) Phosphorylation of the Pol II CTD by KIN28 Enhances BUR1/BUR2 Recruitment and Ser2 CTD Phosphorylation Near Promoters. Mol Cell 33: 752–762 doi:10.1016/j.molcel.2009.02.018. 19328068 10.1016/j.molcel.2009.02.018 PMC2683426

[37] Skaar DA (2002) Characterization of the CTD kinase CTDK-I has identified a role for CTDK-I in pre-mRNA 3“ processing through PTI1p, a novel 3″ cleavage/polyadenylation factor Durham, NC: Duke University.

[38] Boone C, Bussey H, Andrews BJ (2007) Exploring genetic interactions and networks with yeast. Nat Rev Genet 8: 437–449. Available:http://eutils.ncbi.nlm.nih.gov/entrez/eutils/elink.fcgi?dbfrom=pubmed&id=17510664&retmode=ref&cmd=prlinks. 10.1038/nrg2085 17510664

[39] Barbera MA, Petes TD (2006) Selection and analysis of spontaneous reciprocal mitotic cross-overs in Saccharomyces cerevisiae. Proc Natl Acad Sci USA 103: 12819–12824 doi:10.1073/pnas.0605778103. 16908833 10.1073/pnas.0605778103 PMC1550776

[40] Coïc E, Feldman T, Landman AS, Haber JE (2008) Mechanisms of Rad52-independent spontaneous and UV-induced mitotic recombination in Saccharomyces cerevisiae. Genetics 179: 199–211. Available:http://eutils.ncbi.nlm.nih.gov/entrez/eutils/elink.fcgi?dbfrom=pubmed&id=18458103&retmode=ref&cmd=prlinks. 10.1534/genetics.108.087189 PMC2390599 18458103

[41] St Charles J, Hazkani-Covo E, Yin Y, Andersen SL, Dietrich FS, et al (2012) High-resolution genome-wide analysis of irradiated (UV and γ-rays) diploid yeast cells reveals a high frequency of genomic loss of heterozygosity (LOH) events. Genetics 190: 1267–1284 doi:10.1534/genetics.111.137927. 22267500 10.1534/genetics.111.137927 PMC3316642

[42] Lettier G, Feng Q, de Mayolo AA, Erdeniz N, Reid RJD, et al (2006) The role of DNA double-strand breaks in spontaneous homologous recombination in S. cerevisiae. PLoS Genet 2: e194 doi:10.1371/journal.pgen.0020194. 17096599 10.1371/journal.pgen.0020194 PMC1635536

[43] Malone RE (1983) Multiple mutant analysis of recombination in yeast. Mol Gen Genet 189: 405–412. Available:http://eutils.ncbi.nlm.nih.gov/entrez/eutils/elink.fcgi?dbfrom=pubmed&id=22567676&retmode=ref&cmd=prlinks. 10.1007/BF00325902 22567676

[44] Kizer KO, Phatnani HP, Shibata Y, Hall H, Greenleaf AL, et al (2005) A novel domain in Set2 mediates RNA polymerase II interaction and couples histone H3 K36 methylation with transcript elongation. Mol Cell Biol 25: 3305–3316 doi:10.1128/MCB.25.8.3305-3316.2005. 15798214 10.1128/MCB.25.8.3305-3316.2005 PMC1069628

[45] Li M, Phatnani HP, Guan Z, Sage H, Greenleaf AL, et al (2005) Solution structure of the Set2-Rpb1 interacting domain of human Set2 and its interaction with the hyperphosphorylated C-terminal domain of Rpb1. Proc Natl Acad Sci USA 102: 17636–17641 doi:10.1073/pnas.0506350102. 16314571 10.1073/pnas.0506350102 PMC1308900

[46] Li M, Phatnani HP, Greenleaf AL, Zhou P (2006) NMR assignment of the SRI domain of human Set2/HYPB. Journal of biomolecular NMR 36 Suppl 15 doi:10.1007/s10858-005-4690-8. 16435090 10.1007/s10858-005-4690-8

[47] Strahl BD, Grant PA, Briggs SD, Sun Z-W, Bone JR, et al (2002) Set2 is a nucleosomal histone H3-selective methyltransferase that mediates transcriptional repression. Mol Cell Biol 22: 1298–1306. 11839797 10.1128/mcb.22.5.1298-1306.2002 PMC134702

[48] Kim M, Krogan NJ, Vasiljeva L, Rando OJ, Nedea E, et al (2004) The yeast Rat1 exonuclease promotes transcription termination by RNA polymerase II. Nature 432: 517–522 doi:10.1038/nature03041. 15565157 10.1038/nature03041

[49] Ursic D (2004) Multiple protein/protein and protein/RNA interactions suggest roles for yeast DNA/RNA helicase Sen1p in transcription, transcription-coupled DNA repair and RNA processing. Nucleic Acids Res 32: 2441–2452 doi:10.1093/nar/gkh561. 15121901 10.1093/nar/gkh561 PMC419450

[50] Chinchilla K, Rodriguez-Molina JB, Ursic D, Finkel JS, Ansari AZ, et al (2012) Interactions of Sen1, Nrd1, and Nab3 with Multiple Phosphorylated Forms of the Rpb1 C-Terminal Domain in Saccharomyces cerevisiae. Eukaryotic Cell 11: 417–429 doi:10.1128/EC.05320-11. 22286094 10.1128/EC.05320-11 PMC3318303

[51] Bartkowiak B, Liu P, Phatnani HP, Fuda NJ, Cooper JJ, et al (2010) CDK12 is a transcription elongation-associated CTD kinase, the metazoan ortholog of yeast Ctk1. Genes Dev 24: 2303–2316 doi:10.1101/gad.1968210. 20952539 10.1101/gad.1968210 PMC2956209

[52] Berchuk CGARN (2011) Integrated genomic analyses of ovarian carcinoma. Nature 474: 609–615 doi:10.1038/nature10166. 21720365 10.1038/nature10166 PMC3163504

[53] Grasso CS, Wu Y-M, Robinson DR, Cao X, Dhanasekaran SM, et al. (2012) The mutational landscape of lethal castration-resistant prostate cancer. Nature: 1–5. doi:10.1038/nature11125. 10.1038/nature11125 PMC3396711 22722839

[54] Juhlin C, Larsson C, Yakoleva T, Leibiger I, Leibiger B, et al (2006) Loss of parafibromin expression in a subset of parathyroid adenomas. Endocr Relat Cancer 13: 509–523 doi:10.1677/erc.1.01058. 16728578 10.1677/erc.1.01058

[55] Winzeler EA, Liang H, Shoemaker DD, Davis RW (2000) Functional analysis of the yeast genome by precise deletion and parallel phenotypic characterization. Novartis Found Symp 229: 105–9–discussion109–11. 11084935 10.1002/047084664x.ch14

[56] Moreland RB, Nam HG, Hereford LM, Fried HM (1985) Identification of a nuclear localization signal of a yeast ribosomal protein. Proc Natl Acad Sci USA 82: 6561–6565. 3931077 10.1073/pnas.82.19.6561 PMC391249

[57] Lee JM, Greenleaf AL (1989) A protein kinase that phosphorylates the C-terminal repeat domain of the largest subunit of RNA polymerase II. Proc Natl Acad Sci USA 86: 3624–3628. 2657724 10.1073/pnas.86.10.3624 PMC287190

[58] Duina AA, Winston F (2004) Analysis of a mutant histone H3 that perturbs the association of Swi/Snf with chromatin. Mol Cell Biol 24: 561–572. Available:http://eutils.ncbi.nlm.nih.gov/entrez/eutils/elink.fcgi?dbfrom=pubmed&id=14701730&retmode=ref&cmd=prlinks. 10.1128/MCB.24.2.561-572.2004 PMC343804 14701730

[59] Malone RE, Hoekstra MF (1984) Relationships between a hyper-rec mutation (REM1) and other recombination and repair genes in yeast. Genetics 107: 33–48. 6373496 10.1093/genetics/107.1.33 PMC1202313

[60] Lea DE, Coulson CA (1949) The distribution of the numbers of mutants in bacterial populations. J Genet 49: 246–285. 24536673 10.1007/BF02986080

[61] Hall BM, Ma C-X, Liang P, Singh KK (2009) Fluctuation AnaLysis CalculatOR: a web tool for the determination of mutation rate using Luria-Delbruck fluctuation analysis. Bioinformatics 25: 1564–1565. Available:http://bioinformatics.oxfordjournals.org/content/25/12/1564. 10.1093/bioinformatics/btp253 PMC2687991 19369502

