# Supplementary figures and images for: A DNA Damage Response System Associated with the phosphoCTD of Elongating RNA Polymerase II
Source: PLoS One. 2013 Apr 16;8(4):e60909. doi: 10.1371/journal.pone.0060909 (PMC3629013; doi:10.1371/journal.pone.0060909)

Fig. S1

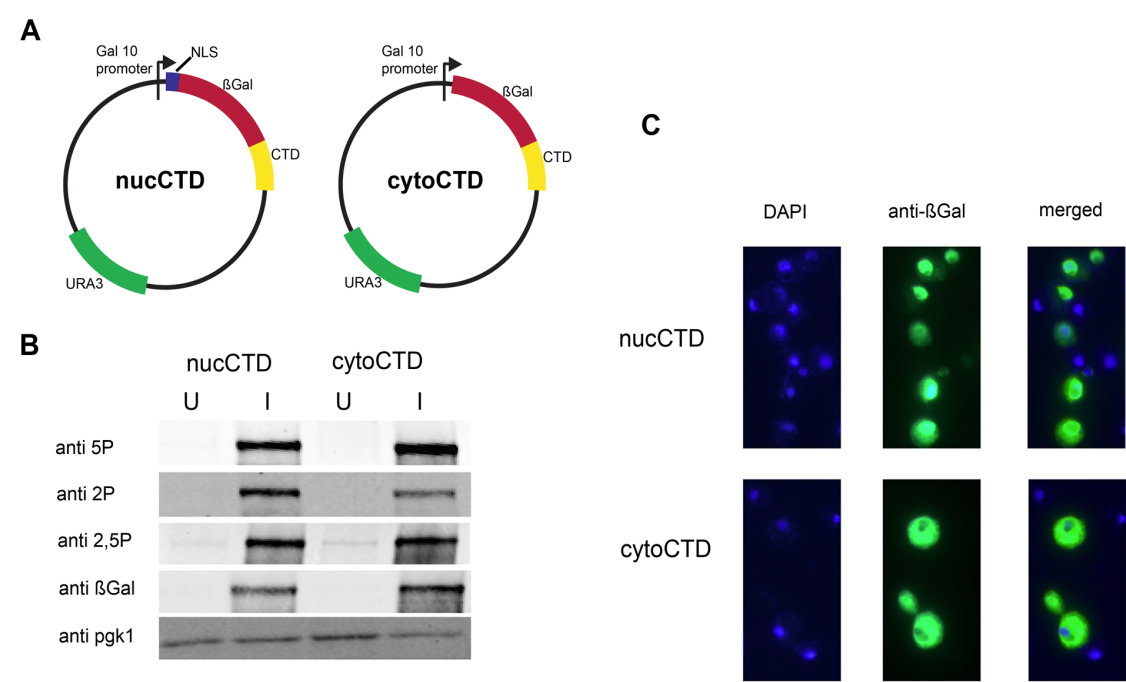

Supplement: Figure S1 — CTD fusion protein constructs, expression, phosphorylation and localization. A. Plasmids for expression of CTD fusion proteins. NLS, Nuclear Localization Signal; ßGal, N-terminal 2/3 of ß-Galactosidase. B. Western Blots of extracts with uninduced (U) or induced (I) CTD fusion proteins. Antibodies against Ser5 phosphorylated (anti 5P), Ser2 phosphorylated (anti 2P), hyper-phosphorylated CTD (anti 2,5P), ß-Galactosidase (anti ßGal), and a loading control (anti pgk1), show that the fusion proteins are phosphorylated. C. Immunoflourescense of strains expressing the fusion proteins. Comparing nuclear staining (DAPI) with fusion protein expression (anti-ßGal) shows that the fusion proteins are properly localized. (PDF) [file pone.0060909.s001.pdf]

Fig. S2

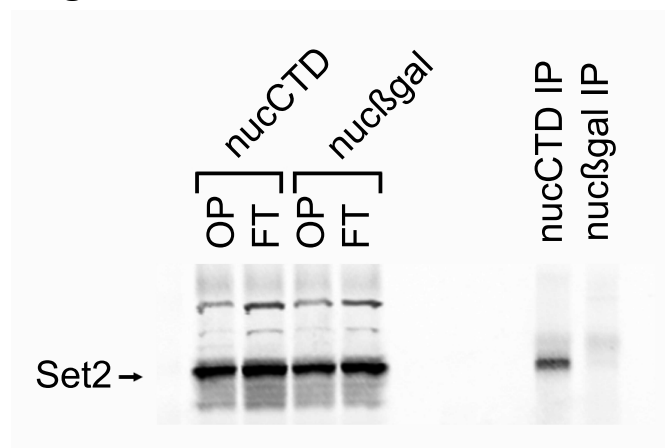

Supplement: Figure S2 — Pull-down of Set2 by CTD fusion proteins. Fusion proteins were expressed in WT yeast cells and pulled down by immunoprecipitation (IP) with an anti ß-Galactosidase antibody. Co-IP of Set2 is illustrated via western blot using an antibody against Set2. Onput (OP) and Flow Through (FT) show Set2 is present in the extract. IP from extract in which nucCTD is expressed shows that Set2 is pulled down by nucCTD fusion protein; however, Set2 is not pulled down by nucßgal fusion protein (lacking a CTD). (PDF) [file pone.0060909.s002.pdf]

Fig. S3

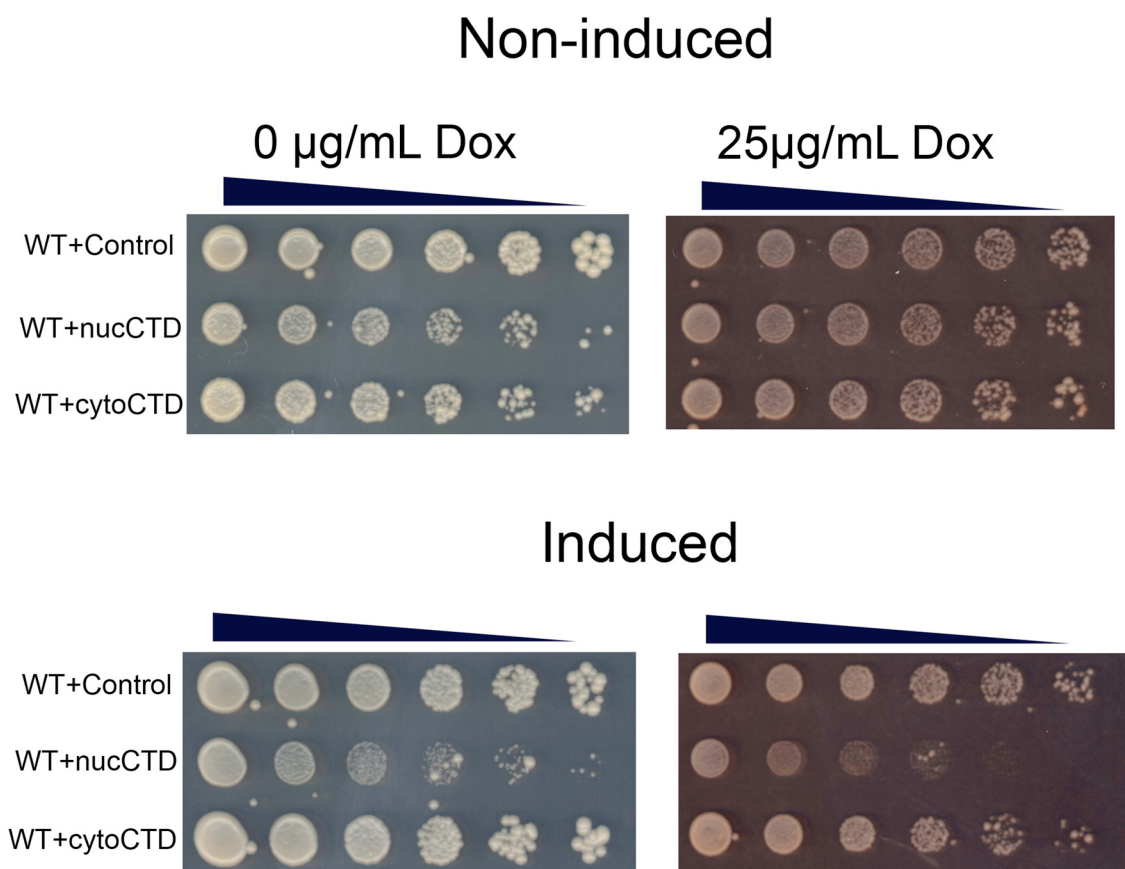

Supplement: Figure S3 — Interfering with binding of PCAPs to the CTD of elongating RNAPII leads to Doxorubicin (DX) sensitivity. NucCTD-, cytoCTD- and empty vector-carrying strains (as in Fig. 1) were spotted in 5-fold serial dilutions on plates containing glucose (Non-induced) or galactose (Induced) and either 0 or 25 µg/ml DX, grown at 30°C for 3 days and photographed. Note that while the nucCTD strain grows slower than the others under inducing conditions, the presence of DX accentuates the difference between it and the other two strains (for nucCTD, colonies are present in all six dilution spots in the absence of DX but are not present in the rightmost two spots in the presence of DX). (PDF) [file pone.0060909.s003.pdf]

Fig. S4

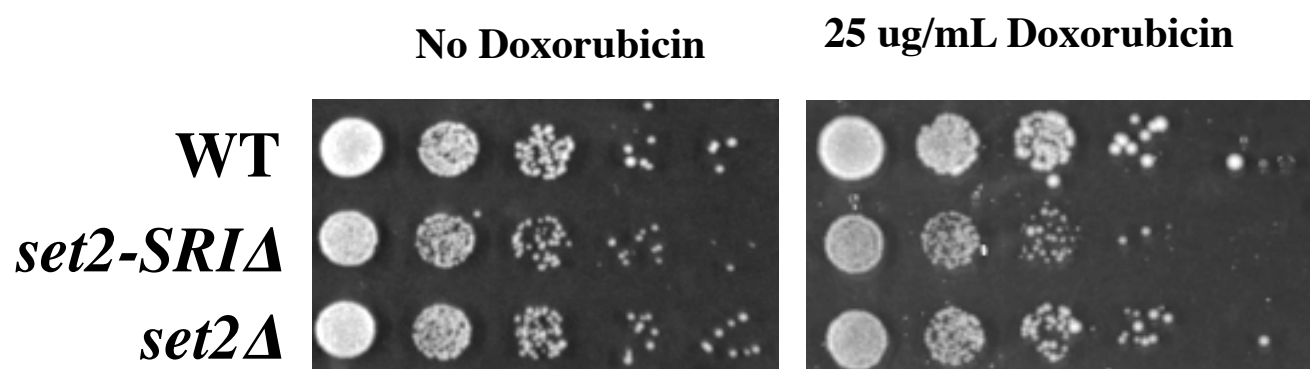

Supplement: Figure S4 — The SRI domain of Set2 is required for damage resistance. Serial dilutions of SET2 WT, ΔSRI and complete gene deletion (set2Δ) strains were spotted on rich (YPD) medium containing either zero or 25 µg/ml DX, grown for 3 days at 30°C and photographed. “No Doxorubicin” results show that very similar numbers of cells were spotted for the three strains and that growth rates are quite similar (size of isolated colonies). (PDF) [file pone.0060909.s004.pdf]
