# Supplementary material for: A DNA Damage Response System Associated with the phosphoCTD of Elongating RNA Polymerase II
Source: PLoS One. 2013 Apr 16;8(4):e60909. doi: 10.1371/journal.pone.0060909 (PMC3629013; doi:10.1371/journal.pone.0060909)
Supplement: Table S1 — ctk1Δ strains are sensitive to DNA damaging agents. (PDF) [file pone.0060909.s005.pdf]

Table S1

ctk1Δ* strains are sensitive to DNA damaging agents			
**agent**		**reduced resistance?**	**references**
DX	doxorubicin	✔	1
HU	hydroxyurea	✔	2
MMS	methylmethane sulfonate	✔	3
UV	ultraviolet radiation	✔	3, 2
4NQO	4-nitroquinoline oxide	✔	3

**ctk1Δ* strains are viable, although they grow slowly and are cold-sensitive.

1. Westmoreland TJ, Wickramasekara SM, Guo AY, Selim AL, Winsor TS, et al. (2009) Comparative genome-wide screening identifies a conserved doxorubicin repair network that is diploid specific in Saccharomyces cerevisiae. PLoS ONE 4: e5830. doi:10.1371/journal.pone.0005830.
2. Ostapenko D, Solomon MJ (2003) Budding yeast CTDK-I is required for DNA damage-induced transcription. Eukaryotic Cell 2: 274–283.
3. Jeong S-J, Kim H-J, Yang Y-J, Seol J-H, Jung B-Y, et al. (2005) Role of RNA polymerase II carboxy terminal domain phosphorylation in DNA damage response. J Microbiol 43: 516–522.
