# Supplementary material for: A DNA Damage Response System Associated with the phosphoCTD of Elongating RNA Polymerase II
Source: PLoS One. 2013 Apr 16;8(4):e60909. doi: 10.1371/journal.pone.0060909 (PMC3629013; doi:10.1371/journal.pone.0060909)
Supplement: Table S2 — CTK1,2,3 show synthetic lethal relationships with HR genes and "DNA-Integrity" genes. (PDF) [file pone.0060909.s006.pdf]

# Table S2

***CTK1,2,3* show synthetic lethal relationships with HR genes and "DNA-Integrity" genes***

| | DNA Integrity genes | CTKn SL |
|---|---|---|
| | ARD1 | CTK2, 3 |
| | ASF1 | CTK1, 2, 3 |
| | BRE1 | CTK1, 2, 3 |
| | CCR4 | CTK1 |
| | CSM1 | CTK1, 2, 3 |
| | CTF18 | CTK1, 2, 3 |
| | CTF8 | CTK1, 2 |
| | DCC1 | CTK1, 2 |
| | DUN1 | CTK1, 2, 3 |
| | ELG1 | CTK1 |
| | HEX3/SLX5 | CTK1, 2, 3 |
| | HIR1 | CTK1, 2, 3 |
| | HIR2 | CTK1, 2, 3 |
| | HPR5/SRS2 | CTK1, 2, 3 |
| | LGE1 | CTK1, 2, 3 |
| | LRS4 | CTK1, 2, 3 |
| | LYS7/CCS1 | CTK1, 2, 3 |
| | MDM39/GET1 | CTK1, 2, 3 |
| | MMS22 | CTK3 |
| HR– | MRE11 | CTK1, 2, 3 |
| | NAT1 | CTK2 |
| | POL32 | CTK3 |
| | POP2/CAF1 | CTK1 |
| | RAD18 | CTK1, 2, 3 |
| | RAD27 | CTK1, 2, 3 |
| | RAD50 | CTK1, 2, 3 |
| | RAD51 | CTK1, 2, 3 |
| HR genes | RAD52 | CTK1, 2, 3 |
| | RAD53 | CTK3 |
| | RAD54 | CTK1, 2, 3 |
| | RAD55 | CTK1, 2, 3 |
| | RAD6 | CTK1, 2, 3 |
| | RMD7/GET2 | CTK1, 2, 3 |
| | RPN4 | CTK1, 2, 3 |
| | RPN10 | CTK3 |
| | SLX4 | CTK1, 3 |
| | TSA1 | CTK2, 3 |
| HR– | XRS2 | CTK1, 2, 3 |

*DNA Integrity genes as defined by Pan et al. (2006). *CELL*, *124*(5), 1069–1081.
