# Supplementary material for: A DNA Damage Response System Associated with the phosphoCTD of Elongating RNA Polymerase II
Source: PLoS One. 2013 Apr 16;8(4):e60909. doi: 10.1371/journal.pone.0060909 (PMC3629013; doi:10.1371/journal.pone.0060909)
Supplement: Table S3 — (PDF) [file pone.0060909.s007.pdf]

Table S3

DNA Integrity Genes	CAR genes that are synthetic lethal (SL) with DNA integrity genes								
ARD1	CTK2, 3	CDC73			RVS161	SAC1	SET2		
ASF1	CTK1, 2, 3	CDC73			RVS161		SET2	UME6	
BRE1	CTK1, 2, 3					SAC1	SET2	UME6	
CCR4	CTK1	CDC73		NOT5	RVS161	SAC1			
CSM1	CTK1, 2, 3								
CTF18	CTK1, 2, 3		CHL1						
CTF8	CTK1, 2		CHL1						
DCC1	CTK1, 2		CHL1				SET2	UME6	
DUN1	CTK1, 2, 3	CDC73							
ELG1	CTK1		CHL1						
HEX3/SLX5	CTK1, 2, 3						SET2		
HIR1	CTK1, 2, 3	CDC73							
HIR2	CTK1, 2, 3								
HPR5/SRS2	CTK1, 2, 3		CHL1						
LGE1	CTK1, 2, 3					SAC1	SET2	UME6	ZUO1
LRS4	CTK1, 2, 3								
LYS7/CCS1	CTK1, 2, 3								
MDM39/GET1	CTK1, 2, 3	CDC73		NOT5	RVS161	SAC1		UME6	
MMS22	CTK3	CDC73							ZUO1
MRE11	CTK1, 2, 3								
NAT1	CTK2	CDC73			RVS161	SAC1	SET2		
POL32	CTK3				RVS161				
POP2/CAF1	CTK1			NOT5	RVS161	SAC1			
RAD18	CTK1, 2, 3	CDC73							
RAD27	CTK1, 2, 3	CDC73	CHL1			SAC1		UME6	
RAD50	CTK1, 2, 3								
RAD51	CTK1, 2, 3								
RAD52	CTK1, 2, 3	CDC73							
RAD53	CTK3								
RAD54	CTK1, 2, 3								
RAD55	CTK1, 2, 3								
RAD6	CTK1, 2, 3				RVS161	SAC1	SET2	UME6	ZUO1
RMD7/GET2	CTK1, 2, 3			NOT5	RVS161	SAC1		UME6	
RPN4	CTK1, 2, 3				RVS161			UME6	
RPN10	CTK3								
SLX4	CTK1, 3								
TSA1	CTK2, 3								
XRS2	CTK1, 2, 3	CDC73							

The 38 DNA integrity genes (Pan, X. et al. CELL 124, 1069–1081 [2006]; and SGD [Saccharomyces Genome Database, <http://www.yeastgenome.org>]) annotated as displaying synthetic lethality with *CTK1, 2* or *3* are listed alphabetically in column 1. *CTKn* SL interactions with genes in column 1 are in column 2. The subsequent columns list SL interactions between each of 8 CAR genes and the genes in column 1 (*HTZ1* interactions were not included). Interaction data are from SGD and we use "SL" to include synthetic fitness/growth defects.
