# Supplementary material for: A DNA Damage Response System Associated with the phosphoCTD of Elongating RNA Polymerase II
Source: PLoS One. 2013 Apr 16;8(4):e60909. doi: 10.1371/journal.pone.0060909 (PMC3629013; doi:10.1371/journal.pone.0060909)
Supplement: Table S4 — Yeast CAR genes, recognized functions, and human orthologs. (PDF) [file pone.0060909.s008.pdf]

Table S4

Yeast **CAR** genes, recognized functions, and human orthologs

CAR gene	recognized function	human ortholog
CDC73	transcription factor (Paf1 complex)	**CDC73**
CHL1	DNA helicase	**DDX11**
CTK1	CTD kinase I, catalytic subunit	**CDK12**
CTK2	CTD kinase I, cyclin-like subunit	**CYCLIN K**
CTK3	CTD kinase I, regulatory (?) subunit	**?**
FUN12	eIF5B	**EIF5B**
GCN5	HAT	**KAT2A**
HOG1	protein kinase	**p38 MAP kinase**
HRR25	casein kinase	**CSNK1D**
HTZ1	histone variant	**H2AFX**
JEM1	chaperone	**DNAJA3**
LIA1	hydroxylase	**DOHH**
MRT4	mRNA turnover	**MRTO4**
NOT5	transcription; RNA degradation	**CNOT3**
PHO2	transcription factor	**PITX3**
PUS1	pseudo-U synthase	**PUS1**
RAI1	RNase subunit; mRNA 5' PPase	**DOM3Z**
RDS2	unk	
RVS161	cytoskeleton; budding	**BIN3**
SAC1	inositol phosphate phosphatase	**SACM1L**
SET2	histone methyltransferase	**SETD2**
SPT7	subunit of SAGA txn complex	**SUPT7L**
UME6	transcription factor	
YPL260w	unk	
ZUO1	chaperone	**DNAJC2**

CAR genes from Table 1, and recognized functions from SGD. Human orthologs from NCBI Entrez Gene, *via* NCBI BLAST, NCBI HOMOLOGENE, ExPASy GPSDB, SMART database, GeneCards.
