# Supplementary material for: A DNA Damage Response System Associated with the phosphoCTD of Elongating RNA Polymerase II
Source: PLoS One. 2013 Apr 16;8(4):e60909. doi: 10.1371/journal.pone.0060909 (PMC3629013; doi:10.1371/journal.pone.0060909)
Supplement: Table S5 — Yeast strains. (PDF) [file pone.0060909.s009.pdf]

Table S5

Yeast Strains

Strain	Genotype	Origin
BY4743	MAT a/α *his3Δ1/his3Δ1 leu2Δ0/leu2Δ0 lys2Δ0/LYS2 MET15/met15Δ0 ura3Δ0/ura3Δ0*	yeast deletion collection
BY4743 *set2Δ*	MAT a/α *his3Δ1/his3Δ1 leu2Δ0/leu2Δ0 lys2Δ0/LYS2 MET15/met15Δ0 ura3Δ0/ura3Δ0 set2Δ::KanMX/set2Δ::KanMX*	yeast deletion collection
BY4743 *set2ΔSRI*	MAT a/α *his3Δ1/his3Δ1 leu2Δ0/leu2Δ0 lys2Δ0/LYS2 MET15/met15Δ0 ura3Δ0/ura3Δ0 set2Δ::KanMX/set2ΔSRI::KanMX*	haploids used in construction from Brian Strahl
BY4743 *SET2/set2Δ* (WT SET2 strain in Chapter 3)	MAT a/α *his3Δ1/his3Δ1 leu2Δ0/leu2Δ0 lys2Δ0/LYS2 MET15/met15Δ0 ura3Δ0/ura3Δ0 set2Δ::KanMX/SET2*	haploids used in construction from Brian Strahl
LRY1443/LRY1444	MAT a/α *ade2-1/ade2-1 can1-100/can1-100 his3-11/his3-11 leu2-3,112/leu2-3,112 myc-SUM1/myc-SUM1 trp1-1/trp1-1 ura3-1/ura3-1* p^{GAS2}*-HIS3/* p^{GAS2}*-HIS3 hht1-hhf1Δ::NatMX hht2-hhf2::HygMX*	haploids used in construction from Laura Rusche
M7/M53 WT recombination strain	MAT a/α *lys2-2 /lys2-1 tyr1-2/tyr1-1 his7-1/his7-2 CAN1/can^r ura3-1/ura3-13 cyh2^r/CYH2 ADE5/ade5 ade2-1/ade2-1 ade6/ADE6 leu1-c/leu1-12 trp5-c/trp5-d met13c*/met13-d*	haploid used in construction made by Robert Malone
M7/M53 *ctk1Δ*	MAT a/α *lys2-2 /lys2-1 tyr1-2/tyr1-1 his7-1/his7-2 CAN1/can^r ura3-1/ura3-13 cyh2^r/CYH2 ADE5/ade5 ade2-1/ade2-1 ade6/ADE6 leu1-c/leu1-12 trp5-c/trp5-d met13c*/met13-d ctk1Δ::KanMX/ctk1Δ::KanMX*	derived from M7/M53
M7/M53 *rvs161Δ*	MAT a/α *lys2-2 /lys2-1 tyr1-2/tyr1-1 his7-1/his7-2 CAN1/can^r ura3-1/ura3-13 cyh2^r/CYH2 ADE5/ade5 ade2-1/ade2-1 ade6/ADE6 leu1-c/leu1-12 trp5-c/trp5-d met13c*/met13-d rvs161Δ::KanMX/rvs161Δ::KanMX*	derived from M7/M53
M7/M53 *set2Δ*	MAT a/α *lys2-2 /lys2-1 tyr1-2/tyr1-1 his7-1/his7-2 CAN1/can^r ura3-1/ura3-13 cyh2^r/CYH2 ADE5/ade5 ade2-1/ade2-1 ade6/ADE6 leu1-c/leu1-12 trp5-c/trp5-d met13c*/met13-d set2Δ::KanMX/set2Δ::KanMX*	derived from M7/M53

All other deletion strains mentioned are diploids from the yeast deletion collection and of the BY4743 background. Therefore, they are isogenic to BY4743 as listed above except the gene of interest is eliminated from both alleles using the KanMX marker.
