# Supplementary material for: A DNA Damage Response System Associated with the phosphoCTD of Elongating RNA Polymerase II
Source: PLoS One. 2013 Apr 16;8(4):e60909. doi: 10.1371/journal.pone.0060909 (PMC3629013; doi:10.1371/journal.pone.0060909)
Supplement: Text S1 — METHODS for Supporting Information. This file contains descriptions of methods for (1) Localization of fusion proteins, (2) Antibodies and Western Blotting, and (3) Immunoprecipitations. (DOCX) [file pone.0060909.s010.docx]

**Text S1.** **METHODS for Supporting Information**

**Localization of fusion proteins**

Plasmid-containing cells were grown to confluency in complete medium minus uracil with glucose as the carbon source (CM+glu-ura) and then were washed in water and resuspended in complete medium minus uracil with galactose as the carbon source (CM+gal-ura) to induce expression of the ßGal-CTD fusion protein. Cells were allowed to grow for six hours and were then fixed in formaldehyde. Cells were prepared as previously described ([1]) and stained with both DAPI and mouse anti-ßgalactosidase (Promega). A goat anti mouse secondary conjugated to Alexa Flour 488 (Invitrogen) was used for visualization of the primary antibody.

**Antibodies and Western Blotting**

Extract Preparation:

Plasmid-bearing cells were grown in 20 mL of CM+glu-ura to saturation. Cells were then washed in H2O and split into two equal portions that were resuspended in 50 mL of either CM+glu-ura or CM+gal-ura and incubated approximately 14 hrs. Cells were then pelleted and washed one time in H2O. Cell pellets were flash frozen by extruding cell pellets into liquid N2. Approximately 0.1 g of frozen cells were weighed out and placed in a 2-mL screw cap microvial (Sarstead). Five hundred µL of 95% ethanol was added to each tube and the samples were broken open by shaking for 5 minutes with zirconia beads in Mini-Beadbeater 16 (Biospec). The entire contents of each microvial (including beads) were placed under vacuum (SpeedVac) until dry. 300 µL 2.5X SDS sample buffer without dye was added to each tube, followed by heating 10 minutes at 98°C, with intermittent vortexing. The resulting SDS sample was collected through a hole that was made in the bottom of the tube by centrifugation into another tube. This spin-through was then centrifuged at max speed in a microfuge (ca. 16,000 X g) for 10 minutes to precipitate any unsolubilized material. The SDS-solubilized portion (supernatant) was placed in another tube, and bromophenol blue was added to 0.02% prior to SDS-PAGE.

Antibodies used in western blotting include: “anti-2,5P,” a rabbit polyclonal antibody made against a CTDK-I phosphorylated yeast CTD fusion protein and affinity purified on a two repeat CTD peptide column with serines 2 and 5 phosphorylated in each repeat; “anti-5P,” rat mAb 3E8 ([2]); “anti-2P,” rat mAb 3E10 (6); mouse anti-ßGal, (Promega Z378B); anti-Pgk1, mouse anti-3-phosphoglycerate kinase (Invitrogen A6457).

**Immunoprecipitations**

Strains containing either the nucCTD plasmid or the nucßGal plasmid were grown in raffinose-containing medium to an A600 of 0.6-0.8. At that point, 40% galactose was added to a final concentration of 2% and the cells were grown for 6 more hours. Cells were collected by centrifugation and frozen in liquid nitrogen as described by Rout and colleagues (7). Extraction buffer (50mM HEPES pH 8.0, 100 mM NaCl, 0.5% NP40, 0.1%PMSF, 1% protease inhibitor [Sigma p8215], 1% phosphatase inhibitor [Sigma p2850]) was also frozen and added to the frozen cells for a total mass of approximately 5g (about 2.5g cells + about 2.5 g frozen buffer). Cells+buffer were homogenized in the frozen state using a Retsch MM400 Ball Mill, essentially as described (7), and the resulting powder was stored at -80°C. Approximately 1g aliquots of cell powder were resuspended in 1.5 mL of extraction buffer in the cold room (all subsequent steps at 4°C unless otherwise noted) and then clarified by centrifugation at 14,000Xg for 10 min at 4°C. Meanwhile, 40 uL of magnetic Protein A/G bead slurry (Dynabeads, Invitrogen) per IP were incubated with 100 uL IgG (0.2 mg/mL anti ßGalactosidase affinity purified by ALG)+ 100 uL PBST for 10 min at room temperature while they rotated end over end. This was then added to the supernatant (WCE) and rocked for 1 hr. The beads were then removed from the slurry using a magnet and were washed three times using 1 mL of wash buffer (50 mM HEPES pH 8.0, 100 mM NaCl, 0.5% NP40). Proteins were eluted off the beads by boiling in 50 uL of 1XSDS sample buffer for 5 minutes.

**REFERENCES** for Supporting Information

1. Pringle JR, Adams AE, Drubin DG, Haarer BK (1991) Immunofluorescence methods for yeast. Meth Enzymol 194: 565–602. Available:http://www.ncbi.nlm.nih.gov/sites/entrez?Db=pubmed&Cmd=Retrieve&list_uids=2005809&dopt=abstractplus.

2. Chapman RD, Heidemann M, Hintermair C, Eick D (2008) Molecular evolution of the RNA polymerase II CTD. Trends Genet 24: 289–296. doi:10.1016/j.tig.2008.03.010.
